# Supported bifunctional thioureas as recoverable and reusable catalysts for enantioselective nitro-Michael reactions

**DOI:** 10.3762/bjoc.12.61

**Published:** 2016-04-01

**Authors:** José M Andrés, Miriam Ceballos, Alicia Maestro, Isabel Sanz, Rafael Pedrosa

**Affiliations:** 1 Instituto CINQUIMA and Departamento de Química Orgánica, Facultad de Ciencias, Universidad de Valladolid, Paseo de Belén 7, 47011-Valladolid, Spain

**Keywords:** bifunctional organocatalysts, organocatalysis, stereoselective nitro-Michael addition, supported catalysts, thioureas

## Abstract

The catalytic activity of different supported bifunctional thioureas on sulfonylpolystyrene resins has been studied in the nitro-Michael addition of different nucleophiles to *trans*-β-nitrostyrene derivatives. The activity of the catalysts depends on the length of the tether linking the chiral thiourea to the polymer. The best results were obtained with the thiourea derived from (L)-valine and 1,6-hexanediamine. The catalysts can be used in only 2 mol % loading, and reused for at least four cycles in neat conditions. The ball milling promoted additions also worked very well.

## Introduction

The use of chiral bifunctional thioureas that allow the simultaneous activation of a electrophile, by hydrogen bonding, and a nucleophile, by deprotonation, plays a major role in the stereoselective formation of C–C bonds in different transformations [1–5]. In these processes one of the major problems is related to the recovering of the catalysts. The support of the small molecules on different materials has been proposed as a solution, including their use in continuous flow processes [6–9]. The most popular supports include nanoparticles [10–12], inorganic solids [13–14], and different polystyrene derivatives [15–20].

Bifunctional thioureas were first supported on PEG [21], and later on different materials such as poly(methylhydrosiloxane) [16], polystyrene [18–22], and magnetic nanoparticles [12]. Cinchona-derived thioureas have been also prepared by co-polymerization of polyfunctionalized thiols with olefins [23].

**Figure 1:**
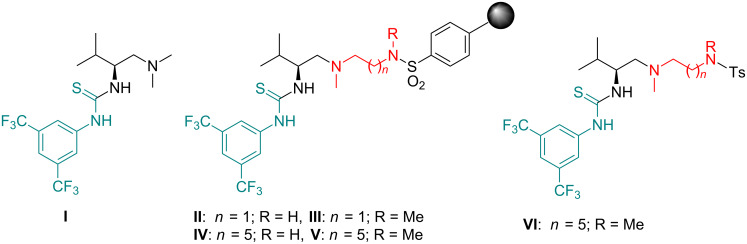
Parent and supported bifunctional thioureas used in this work.

Our interest in the search for novel bifunctional thioureas as organocatalysts [24–27] lead us to consider the preparation of different polymeric materials decorated with chiral bifunctional thioureas looking for a greener process [28], easier recovering and recyclability of the catalyst, and solvent-free reaction conditions. Along these lines, we have recently reported the bottom-up synthesis of polymeric thioureas [29], and the anchorage of (L)-valine-derived thiourea **I** [30] onto sulfonylpolystyrene resin leading to catalysts **II**–**V** (Figure 1), which differ in the length of the diamine linker or in the substitution pattern of the nitrogen in the sulfonamide. These materials, and the related unsupported thiourea **VI**, have been previously tested as excellent organocatalysts in the stereoselective aza-Henry reaction [31]. Now we describe the results obtained in different stereoselective nitro-Michael additions promoted by these materials.

## Results and Discussion

**Scheme 1:**
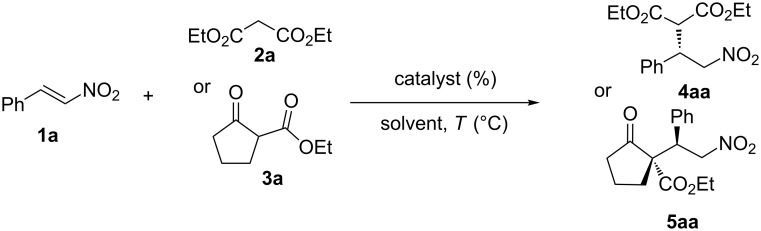
Reaction of nitrostyrene with diethyl malonate and 2-ethoxycarbonyl cyclopentanone.

The ability of the supported catalysts (**II**–**V**) to promote the stereoselective nitro-Michael reaction was first tested in the reaction of *trans*-nitrostyrene (**1a**) with diethyl malonate (**2a**), leading to the enantioenriched addition product **4aa** with a single stereocenter. In order to the creation of two tertiary-quaternary contiguous stereocenters (**5aa**) we also used ethyl 2-oxocyclopentanecarboxylate (**3a**) as nucleophile in neat conditions and in different solvents. For comparative purposes, the same reactions were studied in the presence of unsupported catalysts **I** and **VI**, and the results are summarized in Scheme 1 and Table 1.

**Table 1:** Screening of catalysts and optimization of the reaction conditions for the additions of diethyl malonate and ethyl 2-oxocyclopentanecarboxylate to β-nitrostyrene.

Entry^a^	Catal. (mol %)	Solvent	*T* (°C)	*t* (h)	Product yield^b^ (%)	dr^c^	er^d^

1	**I** (10)	neat	rt	24	**4aa** (80)	–	92:8
2	**II** (10)	neat	rt	120	**4aa** (77)^e^	–	78:22
3	**III** (10)	neat	rt	120	**4aa** (63)^f^	–	85:15
4	**IV** (10)	neat	rt	16	**4aa** (92)	–	91:9
5	**IV** (5)	neat	rt	24	**4aa** (80)	–	90:10
6	**V** (5)	neat	rt	16	**4aa** (92)	–	89:11
7	**VI** (5)	neat	rt	16	**4aa** (100)	–	94:6
8	**I** (5)	neat	rt	1	**5aa** (95)	95:5	95:5
9	**II** (10)	neat	rt	4	**5aa** (100)	89:11	90:10
10	**III** /10)	neat	rt	8	**5aa** (90)	87:13	92:8
11	**IV** (10)	neat	rt	4	**5aa** (100)	88:12	95:5
12	**IV** (5)	neat	rt	7	**5aa** (100)	88:12	95:5
13	**V** (5)	neat	rt	0.5	**5aa** (98)	89:11	95:5
14	**VI** (5)	neat	rt	2	**5aa** (100)	89:11	95:5
15	**V** (2)	neat	rt	1	**5aa** (93)	89:11	95:5
16	**V** (2)	neat	0	24	**5aa** (97)	90:10	94:6
17^g^	**V** (2)	neat	rt	8	**5aa** (86)	88:12	94:6
18^h^	**V** (2)	neat	rt	2.5	**5aa** (83)	88:12	94:6
19	**V** (2)	CH_2_Cl_2_	rt	1	**5aa** (74)	90:10	93:7
20	**V** (2)	PhMe	rt	5	**5aa** (66)	89:11	94:6
21	**V** (2)	THF	rt	1.5	**5aa** (55)	89:11	94:6
22	**V** (2)	MeCN	rt	1.5	**5aa** (79)	89:11	95:5
23	**V** (2)	MeCN	0	8	**5aa** (82)	88:12	95:5
24^g^	**V** (2)	MeCN	rt	8	**5aa** (86)	89:11	92:8

^a^Reaction performed with 2 equiv of nucleophile at room temperature. ^b^Yields refer to isolated compounds. ^c^Determined by ^1^H NMR in the reaction mixture. ^d^Determined by chiral HPLC. ^e^13% of unreacted nitrostyrene was recovered. ^f^15% of unreacted nitrostyrene was recovered. ^g^Reaction performed with 1.1 equiv of ketoester. ^h^Reaction performed with 1.5 equiv of ketoester.

Initially, the reactions were carried out in neat conditions at rt with twofold excess of nucleophile and 10 mol % of catalysts (entries 1–4 and 9–11 in Table 1). As a general trend, the reactions were faster, and much more stereoselective for ketoester **3a** than for diethyl malonate **2a**, and that the difference was specially remarkable when supported ethylenediamine-derived thioureas **II** and **III** were used as catalysts (compare entries 2 and 3 versus 9 and 10 in Table 1). It is noteworthy that the results obtained in the reaction catalyzed by supported thiourea **IV** were better than those observed in the addition catalyzed by the parent thiourea **I** (compare Table 1, entries 1 and 4).

The catalyst loading was decreased to 5 mol % for supported hexanediamine-derived catalyst **IV**, observing that the level of stereoselectivity was maintained for both reactions, although at expenses of slight increasing the reaction time (compare Table 1, entries 4 versus 5, and 11 versus 12). In these conditions, supported catalyst **V**, which differs from **IV** in the substitution pattern of the sulfonamide, was the best catalyst for the addition of both **2a** and **3a** to nitrostyrene, yielding products **4aa** and **5aa**, respectively, in much better yield maintaining the stereoselectivity in shorter reaction time (compare entries 5 versus 6 and 12 versus 13 in Table 1). An increase in both the yield and the enantioselectivity for the reaction of **1a** with **2a** (Table 1, entries 6 and 7), but no differences in the reaction of **1a** with **3a** (Table 1, entries 13 and 14) were observed when the unsupported thiourea **VI**, homologous to **V**, was used as organocatalyst.

Taking the supported thiourea **V** as the catalyst of choice, the effects of the catalyst loading, the temperature, the ratio of nucleophile, and the use of different solvents were studied for the reaction of **1a** and **3a**. Fortunately, the reduction of the amount of catalyst to 2 mol % did not influence the stereoselectivity, and only slightly decreased the yield to 93% (compare Table 1, entries 13 and 15). The reaction also proceeded at 0 °C, leading to the addition product **5aa** in 97% and very good stereoselectivity, but in those conditions the reaction time increased to 24 h (Table 1, entry 16).

The reduction of the amount of the nucleophile to 1.1 equivalents (Table 1, entry 17) or 1.5 equivalents (Table 1, entry 18) had only a moderate effect on the yield of the reaction time, increasing it to 8 h and 2,5 h, respectively. The yield dropped to 55–74%, without change in the stereoselectivity, when the reaction was carried out in less polar solvents such as DCM, toluene, or THF (Table 1, entries 19–21), although both the yield and diastereo- and enantioselectivity were maintained when acetonitrile, a more polar solvent, was used (Table 1, entries 22–24).

**Scheme 2:**
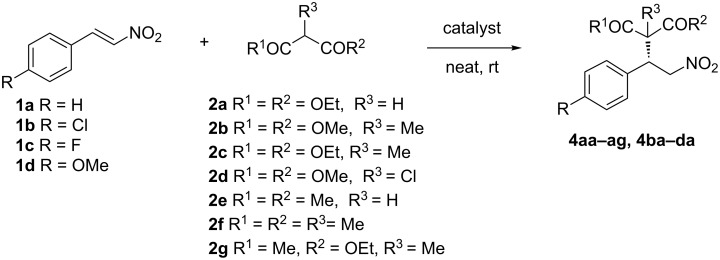
Reaction of nitrostyrenes with malonates and β-diketones.

We next consider the reaction of some 4-substituted nitrostyrenes (**1b–d**) with diethyl malonate (**2a**), and the addition of a range of β-functionalized nucleophiles **2b–g** to **1a** promoted by supported catalysts **IV** (5 mol %) and **V** (2 mol %), respectively (Scheme 2 and Table 2). The results obtained in the reactions of 4-chloro- (**1b**) and 4-fluoronitrostyrene (**1c**) were very similar than those obtained in the reaction with β-nitrostyrene (**1a**), maintaining the yield and the enantioselectivity, although **1c** reacted slowly within 48 h (compare entries 1 and 2 in Table 2 versus entry 5 in Table 1), but the less reactive 4-methoxy derivative **1d** only yielded the addition product in moderate 52% yield after 72 h of reaction (entry 3 in Table 2).

**Table 2:** Addition of malonates and β-diketones to nitrostyrenes catalyzed by **IV** and **V**.

Entry^a^	Reagents	Catal. (mol %)	*t* (h)	Product yield^b^ (%)	dr^c^	er^d^	Config.

1	**1b/2a**	**IV** (5)	6	**4ba** (76)	–	83:17	(*S*)
2	**1c/2a**	**IV** (5)	48	**4ca** (72)	–	86:14	(*S*)
3	**1d/2a**	**IV** (5)	72	**4da** (52)	–	89:11	(*S*)
4	**1a/2b**	**IV** (5)	36	**4ab** (64)	–	92:8	(*R*)
5	**1a/2d**	**IV** (5)	48	**4ad** (74)	–	92:8	(*S*)
6	**1a/2e**	**IV** (5)	5	**4ae** (88)	–	89:11	(*S*)
7	**1a/2f**	**IV** (5)	6	**4af** (70)	–	93:7	(*R*)
8	**1a/2g**	**IV** (5)	7	**4ag** (99)	75:25	92:8	(2*R*,3*R*)
9	**1a/2b**	**V** (2)	2	**4ab** (46)	–	91:9	(*R*)
10	**1a/2c**	**V** (2)	8	**4ac** (47)	–	91:9	(*R*)
11	**1a/2d**	**V** (2)	2	**4ad** (43)	–	91:9	(*S*)
12	**1a/2e**	**V** (5)	2	**4ae** (87)	–	92:8	(*S*)
13	**1a/2f**	**V** (2)	3	**4af** (65)	–	93:7	(*R*)
14	**1a/2g**	**V** (2)	6	**4ag** (81)	74:26	94:6	(2*R*,3*R*)

^a^Reaction performed with 2 equiv of nucleophile. ^b^Yields determined after chromatographic purifications. ^c^Diastereomeric excess determined by ^1^H NMR of the crude reaction mixture. ^d^Enantiomeric ratio determined by chiral HPLC analysis.

The reaction of different nucleophiles (**2a–g**) with **1a** catalyzed by **IV** (5 mol %) also worked well in terms of enantioselectivity, maintaining the er near constant around or higher than 90:10, although the stereoselectivity for the reaction leading to **4ag**, with two contiguous tertiary–quaternary stereocenters was only moderate (dr 75:25, entry 8 in Table 2). The main difference in those additions was related with the reaction time, because the less reactive malonates (**2b** and **2d**) reacted slower than the acetylacetone (**2e** and **2f**) or ethyl acetoacetate (**2g**) derivatives.

Similar results were obtained when the reaction was run in the presence of only 2 mol % of catalyst **V** (entries 9–14 in Table 2). The level of stereoselectivity was maintained, including the diastereoselectivity for the reaction of **1a** with **2g** (dr 74:26), but the yields for the reactions with 2-substituted malonates (**2b–d**) were only moderate (entries 9–11 in Table 2).

**Scheme 3:**
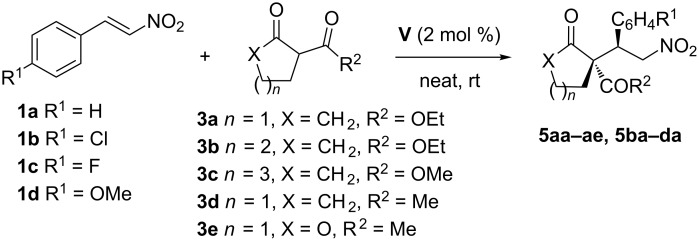
Reaction of nitrostyrenes with β-keto esters and β-dicarbonyl compounds.

Next, we extend the study to the reaction of different β-functionalized cycloalkanones (**3a–c**), 2-acetylcyclopentanone (**3d**), and 2-acetyl-γ-lactone (**3e**) with nitrostyrene derivatives **1a–d** (Scheme 3 and Table 3). The electronic nature of the nitroolefins only affects the yield and the reaction time, being longer as the donating effect of the substituent increases, but maintaining good diastereoselectivity and excellent enantioselectivity for all reactions (entries 2–4, Table 3).

**Table 3:** Reaction of nitrostyrenes with β-substituted cycloalkanones catalyzed by **V**.

Entry^a^	Reagents	Catal. (mol %)	*t* (h)	Product Yield^b^ (%)	dr^c^	er^d^	Config.

1	**1a/3a**	**V** (5)	0.5	**5aa** (98)	89:11	95:5	(*S,R*)
2	**1b/3a**	**V** (2)	1	**5ba** (84)	90:10	94:6	(*S,R*)
3	**1c/3a**	**V** (2)	5	**5ca** (76)	89:11	93:7	(*S,R*)
4	**1d/3a**	**V** (2)	9	**5da** (74)	89:11	95:5	(*S,R*)
5	**1a/3a**	**V** (2)	1	**5aa** (93)	89:11	95:5	(*S,R*)
6	**1a/3b**	**V** (2)	96	**5ab** (63)	88:12	96:4	(*S,R*)
7	**1a/3b**	**V** (5)	10	**5ab** (62)	88:12	96:4	(*S,R*)
8	**1a/3c**	**V** (2)	24	**5ac** (73)	82:18	96:4	(*S,R*)
9	**1a/3d**	**V** (2)	2	**5ad** (92)	83:17	93:7	(*R,R*)
10	**1a/3e**	**V** (2)	1	**5ae** (89)	70:30	92:8	(*R,R*)
11^e^	**1a/3a**	**V** (5)	1	**5aa** (81)	88:12	94:6	(*S,R*)
12^f^	**1a/3a**	**V** (5)	1	**5aa** (76)	88:12	95:5	(*S,R*)
13^g^	**1a/3a**	**V** (5)	2	**5aa** (82)	88:12	94:6	(*S,R*)

^a^Reaction performed with 2 equiv of nucleophile. ^b^Yields determined after chromatographic purifications. ^c^Diastereomeric excess determined by ^1^H NMR of the crude reaction mixture. ^d^Enantiomeric ratio determined by chiral HPLC analysis. ^e^Second cycle for entry 1 by using only 1.5 equivalents of nucleophile. ^f^Third cycle for entry 1 by using only 1.5 equivalents of nucleophile. ^g^Fourth cycle for entry 1 by using only 1.5 equivalents of nucleophile.

With respect to the nucleophile, it is noteworthy that there seems to be a correlation: With growing size of the cycloalkanone an increase of the reaction time and a decrease of the yield can be observed. Cycloheptanone **3c**, and specially cyclohexanone **3b** reacted much more slowly than cyclopentanone derivative **3a** (Table 3, entries 5, 6, and 8), although the reaction time could be reduced from 96 h to 10 h in the reaction of **1a** with **3b** by increasing the catalyst loading from 2 mol % to 5 mol % (Table 3, entry 7). It is also noteworthy that 2-acetyl-γ-lactone (**3e)** reacted very well with **1a**, leading to **5ae** in excellent yield and enantioselectivity, but only moderate diastereoselectivity (dr 70:30, Table 3, entry 10).

To test the recyclability of catalyst **V** we choose the reaction of **1a** with **3a** in neat conditions and 5 mol % of catalyst as model. Once the reaction had finished (TLC), the catalyst was recovered by filtration, and after washing with methanol and drying to constant weight, the catalyst was used in the next cycle (entries 1 and 11–13 in Table 3). Fortunately, catalyst **V** can be reused for at least four cycles yielding **5aa** in high yields (76–82%), and maintaining both the diastereo- and enantioselectivity.

**Scheme 4:**
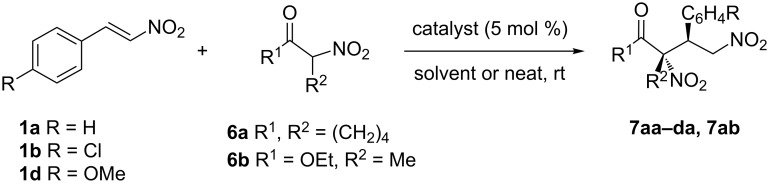
Reaction of nitrostyrenes with α-nitrocyclohexanone and ethyl α-nitropropionate.

Finally, α-nitrocyclohexanone (**6a**) and ethyl α-nitropropionate (**6b**) were included in the screening process in order to compare the results obtained with more acidic nucleophiles with those obtained with β-dicarbonyl derivatives. The reactions were carried out under different conditions by varying the solvent, the temperature and using unsupported thioureas **I** and **VI**, and **V** as an example of a supported one (Scheme 4). The most significant difference between the reactions of nitroketone and nitroester refers to the stereoselectivity in both processes. The addition of nitroketone was totally diastereoselective and highly enantioselective (entries 1–12 in Table 4), whereas both the diastero- and enantioselectivities were moderate for the addition of nitroester (Table 4, entries 13–15). Additionally, supported catalyst **V** was able to promote more enantioselective transformations than unsupported catalyst **I** (compare Table 4, entry 13 versus 15). The decrease of the temperature slows down the reaction, increasing the reaction time from 1 h to 6 h for the reaction of **1a** with **6b**, catalyzed by **I** (Table 4, entries 13 and 14), and from 24 h to 48 h for the addition of **6a** to **1a** catalyzed for **VI** (Table 4, entries 7 and 8), but maintaining the diastereoselectivity and slightly increasing the enantioselectivity. The donor character of the substituent in the nitrostyrene derivative plays an important role in the process, increasing the reaction time, and decreasing the yield (compare Table 4, entries 5, 9, and 11).

**Table 4:** Reactions of nitrostyrenes with α-nitrocyclohexanone and ethyl α-nitropropionate.

Entry^a^	Reagents	Catal. (mol %)	*t* (h)	Solvent	Product yield^b^ (%)	dr^c^	er^d^

1	**1a/6a**	**I** (5)	3	DCM	**7aa** (62)	>98:<2	84:16
2	**1a/6a**	**I** (5)	1.5	neat	**7aa** (65)	>98:<2	90/10
3^e^	**1a/6a**	**I** (5)	1	neat	**7aa** (70)	>98:<2	90:10
4	**1a/6a**	**V** (5)	48	DCM	**7aa** (60)	>98:<2	90:10
5	**1a/6a**	**V** (5)	12	MeCN	**7aa** (77)	>98:<2	91:9
6^e^	**1a/6a**	**V** (5)	12	neat	**7aa** (85)	>98:<2	94:6
7	**1a/6a**	**VI** (5)	24	DCM	**7aa** (64)	>98:<2	85:15
8^f^	**1a/6a**	**VI** (5)	48	DCM	**7aa** (52)	>98:<2	92:8
9	**1b/6a**	**V** (5)	8	MeCN	**7ba** (91)	>98:<2	91:9
10^e^	**1b/6a**	**V** (5)	48	neat	**7ba** (48)^g^	>98:<2	90:10
11	**1d/6a**	**V** (5)	24	MeCN	**7da**(73)	>98:<2	89:11
12^e^	**1d/6a**	**V** (5)	360	neat	**7da**(50)^h^	>98:<2	87:13
13	**1a/6b**	**I** (5)	1	neat	**7ab** (75)	74:26	69:31
14^f^	**1a/6b**	**I** (5)	6	neat	**7ab** (77)	75:25	72:28
15	**1a/6b**	**V** (5)	5	neat	**7ab** (90)	76:24	74:26

^a^Reaction performed with 1.5 equiv of nucleophile. ^b^Yields determined after chromatographic purifications. ^c^Diastereomeric excess determined by ^1^H NMR of the crude reaction mixture (>98:<2 means that a single diastereomer was detected). ^d^Enantiomeric ratio determined by chiral HPLC analysis. ^e^Ball mill conditions. ^f^The reaction was carried out at −20 °C. ^g^37% of 2-nitrocyclohexanone was recovered unreacted. ^h^30% of 2-nitrocyclohexanone was recovered unreacted.

The recent interest in alternative activation modes [32] for promoting C–C bond formations, led us to consider the catalytic addition of α-nitrocyclohexanone (**6a**) to nitrostyrene derivatives **1a**, **1b**, and **1d** under ball milling conditions [33]. We chose these reactions for comparative purposes because they have been previously reported [34]. Although the experimental conditions have not been optimized, we observed that both the unsupported (**I**) and supported (**V**) catalysts worked very well in the reaction of **1a** with **6a** yielding the addition product **7aa** in excellent yields and stereoselectivity (entries 3 and 6 in Table 4). On the contrary, the reactions of **6a** with **1b** and **1d** are very slow and 37% and 30% of the nucleophile were recovered unchanged after 48 h and 360 h of reaction, respectively, although maintaining the stereoselectivity (Table 4, entries 10 and 12). The improvement of that process is under study.

## Conclusion

In summary, supported bifunctional thioureas on sulfonylpolystyrene are able to promote highly stereoselective nitro-Michael reactions with different nucleophiles. The activity of the catalysts varies with the length of the tether between the polymer and the thiourea framework, and the best results were obtained by using catalysts **IV** and **V**, derived from 1,6-hexanediamine. The reactions work well by using only 2 mol % of catalyst loading in neat conditions, and the catalysts can easily be recovered and reused for four cycles. The results obtained with the described catalysts are similar to those previously reported by using bottom-up synthesized materials prepared by co-polymerization of monomeric thioureas as organocatalysts [29]. Catalyst **V** has been also used in the addition of α-nitrocyclohexanone (**6a**) to β-nitrostyrene (**1a**) under solvent-free conditions in a ball mill providing the addition product **7aa** in excellent yield, total diastereoselectivity, and very good enantioselectitvity.

## Experimental

### General remarks

^13^C NMR (126 MHz) and ^1^H NMR (500 MHz) spectra were recorded in CDCl_3_ as the solvent. Chemical shifts for carbons are reported in ppm from TMS and are referenced to the carbon resonance of the solvent. Chemical shifts for protons are reported in ppm from TMS with the residual CHCl_3_ resonance as internal reference. Data are reported as follows: chemical shift, multiplicity (s = singlet, d = doublet, t = triplet, q = quartet, m = multiplet, br = broad), coupling constants in Hertz, and integration.

Flash chromatography was carried out using silica gel (230–240 mesh). TLC analysis was performed on glass-backed plates coated with silica gel 60 and an F_254_ indicator, and visualized by either UV irradiation or by staining with phosphomolybdic acid solution. Specific rotations were measured on a digital polarimeter using a 5 mL cell with a 1 dm path length, and a sodium lamp, and the concentration is given in g per 100 mL. Chiral HPLC analysis was performed by using Daicel Chiralcel OD or Chiralpak AD-H, analytical columns (250 × 4.6 mm) by using mixture of *n*-hexane/isopropanol as eluent. UV detection was monitored at 220 or at 254 nm. ESI mass spectra were obtained on an Agilent 5973 inert GC/MS system. Commercially available organic and inorganic compounds were used without further purification. Solvents were dried and stored over microwave–activated 4 Å molecular sieves. Supported thioureas **II**–**V** and unsupported thioureas **I** and **VI** were prepared according to reported procedures [30–31]. Racemic reference samples were prepared by using DABCO (5 mol %) following the same procedure as described below.

**General procedure for the nitro-Michael reaction using homogeneous catalysts (I and VI).** The reactions were carried out as previously described [29]. To a mixture of nitrostyrene (0.3 mmol) and catalyst (0.015 mmol, 0.05 equiv), the 1,3-dicarbonyl compound (0.6 mmol, 2 equiv) was added and the reaction mixture was stirred at rt in a Wheaton vial until consumption of the starting material (monitoring by TLC). The reaction mixture was purified by column chromatography to afford the Michael product. The *anti*- and *syn*-isomers of the Michael products were not separated by column chromatography. The diastereomeric ratio was determined by ^1^H NMR spectroscopy of the purified product.

**General procedure for the nitro-Michael reaction using inmobilized catalysts (II, III, IV and V)** [29]. To a mixture of β-nitrostyrene (0.3 mmol) and catalyst (0.015 mmol, 0.05 equiv), 1,3-dicarbonyl compound (0.6 mmol, 2 equiv) was added and the reaction mixture was stirred at rt in a Wheaton vial until consumption of the starting material (monitored by TLC). The catalyst was filtered off and washed with MeOH (3 × 1 mL). The solvent was removed under reduced pressure, and the residue was purified by column chromatography. The *anti*- and *syn*-isomers of the Michael products were not separated by column chromatography. The diastereomeric ratio was determined by ^1^H NMR spectroscopy of the purified product.

**Recyclability of the supported thiourea catalysts in nitro-Michael reaction.** The supported catalysts were recovered from the reaction mixtures by filtration, thoroughly washed with methanol, dried and reused in the next cycle.

### General procedure for the Michael addition of 2-nitrocyclohexanone to β-nitrostyrene using immobilized catalyst **VI**

**Method A, in organic solvent:** The reactions were carried out as previously described [29]. To a stirred solution of 2-nitrocyclohexanone (43 mg, 0.3 mmol) and nitroalkene (0.45 mmol, 1.5 equiv) in an adequate solvent (0.4 mL), catalyst **VI** (15 mg, 0.015 mmol, 0.05 equiv) was added and the reaction mixture was stirred at room temperature in a Wheaton vial until the reaction was finished (TLC). The catalyst was filtered off and washed with DCM (3 × 1 mL). The solvent was removed under reduced pressure, the crude mixture subjected to flash chromatography to afford the Michael adduct. The diastereomeric ratio was determined by ^1^H NMR spectroscopy of the purified product. The enantiomeric excess was determined by chiral-phase HPLC analysis using mixtures of hexane/isopropanol as eluent.

**Method B, under ball-milling conditions:** Catalyst **VI** (15 mg, 0.015 mmol, 0.05 equiv), 2-nitrocyclohexanone (43 mg, 0.3 mmol) and nitroalkene (0.45 mmol, 1.5 equiv) were transferred to a clean, dry ball milling vessel (cylinder of 5 mL) loaded with two grinding balls with a 7 mm diameter. The vessel was placed in a Mixer Mill MM 200 and the mixture was milled at 5 Hz of vibrational frequency at room temperature until consumption of the starting material (monitored by TLC). The vessel and the balls were washed with CH_2_Cl_2_, the catalyst was filtered off and washed with CH_2_Cl_2_ and methanol. The resulting solution was concentrated in vacuo, and the product was purified by flash chromatography. The diastereomeric ratio was determined by ^1^H NMR spectroscopy of the purified product. The enantiomeric excess was determined by chiral-phase HPLC analysis using mixtures of hexane/isopropanol as eluent.

## Supporting Information

File 1 Physical and spectral data for all the compounds. Copies of ^1^H, ^13^C NMR spectra, and HPLC traces for all compounds synthesized.
