# Supplementary material for: Supported bifunctional thioureas as recoverable and reusable catalysts for enantioselective nitro-Michael reactions
Source: Beilstein J Org Chem. 2016 Apr 1;12:628–35. doi: 10.3762/bjoc.12.61 (PMC4902052; doi:10.3762/bjoc.12.61)
Supplement: File 1 — Physical and spectral data for all the compounds. Copies of 1H, 13C NMR spectra, and HPLC traces for all compounds synthesized. [file Beilstein_J_Org_Chem-12-628-s001.pdf]

Supporting Information

for

Supported bifunctional thioureas as recoverable and reusable catalysts for enantioselective nitro-Michael reactions

José M. Andrés*, Miriam Ceballos, Alicia Maestro, Isabel Sanz and Rafael Pedrosa*

Address: Instituto CINQUIMA and Departamento de Química Orgánica, Facultad de Ciencias, Universidad de Valladolid, Paseo de Belén 7, 47011-Valladolid, Spain

Email: Rafael Pedrosa - pedrosa@qo.uva.es

*Corresponding author

Physical and spectral data for all the compounds. Copies of ^{1}H, ^{13}C NMR spectra, and HPLC traces for all compounds synthesized

Table of contents

1. Compound characterization data ………………………………………………..… S2

2. NMR spectra for new compounds ………..…………………………….…... S7

3. HPLC profiles of the nitro-Michael products …………………………..… S8

4. References ……………………………………………………………….…. S30

1. Compound characterization data

(S)-Diethyl 2-(2-nitro-1-phenylethyl)malonate (4aa).[1] Colorless solid. **^{1}H NMR** (500 MHz, $CDCl_3$) δ 1.05 (t, *J* = 7.1 Hz, 3H, $C\underline{H}_3$), 1.27 (t, *J* = 7.1 Hz, 3H, $C\underline{H}_3$), 3.82 (d, *J* = 9.3 Hz, 1H, $C\underline{H}CO_2Et$), 4.01 (q, *J* = 7.1 Hz, 2H, $C\underline{H}_2O$), 4.23 (m, 3H, $C\underline{H}_2O$ and $C\underline{H}$), 4.86 (dd, *J* = 13.1, 9.2 Hz, 1H, $C\underline{H}HNO_2$), 4.92 (dd, *J* = 13.1, 4.8 Hz, 1H, $CH\underline{H}NO_2$), 7.23-7.33 (m, 5H, $\underline{H}$ar). **HPLC** (Chiralpak AD-H, *n*-hexane/2-propanol = 80/20, 1.0 mL/min, λ = 220 nm); t_R = 10.0 min (minor, *R*), 24.6 min (major, *S*).

(*R*)-Dimethyl 2-methyl-2-(2-nitro-1-phenylethyl)malonate (4ab).[1] Colorless solid. **^{1}H NMR** (500 MHz, $CDCl_3$) δ 1.36 (s, 3H, $C\underline{H}_3$), 3.74 (s, 3H, $C\underline{H}_3O$), 3.79 (s, 3H, $C\underline{H}_3O$), 4.19 (dd, *J* = 9.9, 4.4 Hz, 1H, $C\underline{H}$), 5.05 (m, 2H, $C\underline{HH}NO_2$), 7.15-7.18 (m, 2H, $\underline{H}$ar), 7.27–7.32 (m, 3H, $\underline{H}$ar). **HPLC** (Chiralpak AD-H, *n*-hexane/2-propanol = 95/5, 0.8 mL/min, λ = 220 nm); t_R = 15.0 min (major, *R*), 16.3 min (minor, *S*).

(*R*)-Diethyl 2-methyl-2-(2-nitro-1-phenylethyl)malonate (4ac).[2] Colorless solid. **^{1}H NMR** (500 MHz, $CDCl_3$) δ 1.26 (t, *J* = 7.2 Hz, 3H, $C\underline{H}_3CH_2$); 1.29 (t, *J* = 7.2 Hz, 3H, $C\underline{H}_3CH_2$), 1.34 (s, 3H, $C\underline{H}_3$), 4.18 (m, 3H, CH_2 and $C\underline{H}$), 4.26 (q, *J* = 7.1 Hz, 2H, $C\underline{H}_2$), 5.05 (m, 2H, $C\underline{H}_2NO_2$), 7.17-7.21 (m, 2H, $\underline{H}$ar), 7.28-7.34 (m, 3H, $\underline{H}$ar). **HPLC** (Chiralpak AD-H, *n*-hexane/2-propanol = 98/2, 0.5 mL/min, λ = 220 nm); t_R = 18.9 min (minor, *S*), 20.8 min (major, *R*).

(*S*)-Dimethyl 2-chloro-2-(2-nitro-1-phenylethyl)malonate (4ad).[1] Colorless solid. **^{1}H NMR** (500 MHz, $CDCl_3$) δ 3.60 (s, 3H, $C\underline{H}_3$), 3.86 (s, 3H, $C\underline{H}_3$), 4.64 (dd, *J* = 10.3, 3.5 Hz, 1H, $C\underline{H}$), 5.01 (dd, *J* = 13.6, 10.3 Hz, 1H, $C\underline{H}HNO_2$), 5.22 (dd, *J* = 13.6, 3.5 Hz, 1H, $CH\underline{H}NO_2$), 7.22–7.41 (m, 5H, $\underline{H}$ar). **HPLC** (Chiralcel OD, *n*-hexane/2-propanol = 90/10, 1.0 mL/min, λ = 220 nm); t_R = 11.3 min (major, *S*), 18.1 min (minor, *R*).

(*S*)-3-(2-Nitro-1-phenylethyl)pentane-2,4-dione (4ae).[3] Colorless solid. **^{1}H NMR** (500 MHz, $CDCl_3$) δ 1.92 (s, 3H, $C\underline{H}_3$), 2.27 (s, 3H, $C\underline{H}_3$), 4.22 (m, 1H, $C\underline{H}$), 4.37 (d, *J* = 10.8 Hz, 1H, $C\underline{H}COMe$), 4.59 (dd, *J* = 11.3, 3.9 Hz, 1H, $C\underline{H}HNO_2$), 4.63 (dd, *J* = 11.3, 6.8 Hz, 1H, $CH\underline{H}NO_2$), 7.15–7.17 (m, 2H, $\underline{H}$ar), 7.24–7.33 (m, 3H, $\underline{H}$ar). **HPLC** (Chiralpak AD–H, *n*-hexane/2-propanol = 85/15, 1.0 mL/min, λ = 220 nm); t_R = 9.5 min (major, *S*), 12.2 min (minor, *R*).

(*R*)-3-Methyl-3-(2-nitro-1-phenylethyl)pentane-2,4-dione (4af). Colorless solid. ^{1}**H NMR** (500 MHz, $CDCl_3$) δ 1.39 (s, 3H, $C\underline{H}_3$), 2.00 (s, 3H, $C\underline{H}_3CO$), 2.06 (s, 3H, $C\underline{H}_3CO$), 4.19 (dd, *J* = 11.0, 3.5 Hz, 1H, $C\underline{H}$), 4.75 (dd, *J* = 13.4, 3.6 Hz, 1H, $C\underline{H}HNO_2$), 4.85 (dd, *J* = 13.4, 11.1 Hz, 1H, $CH\underline{H}NO_2$), 7.14-7.16 (m, 2H, $\underline{H}$ar), 7.24-7.30 (m, 3H, $\underline{H}$ar). ^{13}**C NMR** (75 MHz, $CDCl_3$) δ 18.3 ($\underline{C}H_3$), 27.1, 28.8 ($\underline{C}H_3CO$), 47.4 ($\underline{C}H$), 67.7 ($\underline{C}$), 76.8 ($\underline{C}H_2$), 128.4, 128.8, 129.2 ($\underline{C}$Har), 135.0 ($\underline{C}$ar), 205.6, 208.0 ($\underline{C}O$). IR (ATR): 2987, 1714, 1698, 1551, 1358, 1207, 1087, 962, 705 cm^{-1}. **HRMS** calcd. for $C_{14}H_{17}NO_4$ + Na: 286.1050; found: 286.1053. $[\alpha]_D^{23}$ = +29.0 (c = 0.6, $CHCl_3$) for er 93:7. **HPLC** (Chiralpak AD–H, *n*-hexane/2-propanol = 95/5, 1.0 mL/min, λ = 210 nm); t_R = 17.0 min (minor, *S*), 18.6 min (major, *R*).

(2*R*,3*R*)-Ethyl 2-acetyl-2-methyl-4-nitro-3-phenylbutanoate (4ag).[4] Colorless solid. Major diastereoisomer. ^{1}**H NMR** (500 MHz, $CDCl_3$) δ 1.20 (t, *J* = 7.2 Hz, 3H, $C\underline{H}_3CH_2$), 1.44 (s, 3H, $C\underline{H}_3$), 2.12 (s, 3H, $C\underline{H}_3CO$), 3.98–4.17 (m, 2H, $C\underline{H}_2CH_3$), 4.23 (m, 1H, $C\underline{H}Ph$), 4.94–4.97 (m, 2H, $C\underline{HH}NO_2$), 7.20–7.24 (m, 2H, $\underline{H}$ar), 7.26–7.31 (m, 3H, $\underline{H}$ar). **HPLC** (Chiralcel OD, *n*-hexane/2-propanol = 90/10, 0.8 mL/min, λ = 220 nm): t_R (major diastereoisomer) =13.6 min (major, 2*R*,3*R*), 19.6 min (minor, 2*S*,3*S*).

(*S*)-Diethyl 2-(1-(4-chlorophenyl)-2-nitroethyl)malonate (4ba).[1] Colorless solid. ^{1}**H NMR** (500 MHz, $CDCl_3$) δ 1.06 (t, *J* = 7.2 Hz, 3H, $C\underline{H}_3$), 1.24 (t, *J* = 7.1 Hz, 3H, $C\underline{H}_3$), 3.76 (d, *J* = 9.2 Hz, 1H, $C\underline{H}CO_2Et$), 4.01 (q, *J* = 7.1 Hz, 2H, $C\underline{H}_2O$), 4.20 (m, 3H, $C\underline{H}_2O$ and $C\underline{H}$), 4.81 (dd, *J* = 13.2, 9.3 Hz, 1H, $C\underline{H}HNO_2$), 4.89 (dd, *J* = 13.2, 4.8 Hz, 1H, $CH\underline{H}NO_2$), 7.17 (d, *J* = 8.5 Hz, 2H, $\underline{H}$ar), 7.28 (d, *J* = 8.5 Hz, 2H, $\underline{H}$ar). **HPLC** (Chiralpak AD–H, *n*-hexane/2-propanol = 70/30, 1.0 mL/min, λ = 220 nm); t_R = 9.4 min (minor, *R*), 25.0 min (major, *S*).

(*S*)-Diethyl 2-(1-(4-fluorophenyl)-2-nitroethyl)malonate (4ca).[2] Colorless solid. ^{1}**H NMR** (500 MHz, $CDCl_3$) δ 1.09 (t, *J* = 7.2 Hz, 3H, $C\underline{H}_3$), 1.28 (t, *J* = 7.2 Hz, 3H, $C\underline{H}_3$), 3.79 (d, *J* = 9.3 Hz, 1H, $C\underline{H}CO_2Et$), 4.04 (q, *J* = 7.1 Hz, 2H, $C\underline{H}_2O$), 4.24 (m, 3H, $C\underline{H}_2O$ and $C\underline{H}$), 4.83 (dd, *J* = 13.1, 9.4 Hz, 1H, $C\underline{H}HNO_2$), 4.92 (dd, *J* = 13.1, 4.7 Hz, 1H, $CH\underline{H}NO_2$), 7.02 (m, 2H, $\underline{H}$ar), 7.24 (m, 2H, $\underline{H}$ar). **HPLC** (Chiralpak AD–H, *n*-hexane/2-propanol = 80/20, 1.0 mL/min, λ = 220 nm); t_R = 12.5 min (minor, *R*), 51.6 min (major, *S*).

(*S*)-Diethyl 2-(1-(4-methoxyphenyl)-2-nitroethyl)malonate (4da).[1] Colorless solid. **^{1}H NMR** (500 MHz, $CDCl_3$) δ 1.08 (t, *J* = 7.1 Hz, 3H, CH_3), 1.27 (t, *J* = 7.1 Hz, 3H, CH_3), 3.78 (s, 3H, CH_3O), 3.79 (d, *J* = 10.0 Hz, 1H, $CHCO_2Et$), 4.02 (q, *J* = 7.1 Hz, 2H, CH_2O), 4.22 (m, 3H, CH_2O and CH), 4.82 (dd, *J* = 12.9, 9.3 Hz, 1H, $CHHNO_2$), 4.89 (dd, *J* = 12.9, 4.8 Hz, 1H, $CHHNO_2$), 6.84 (d, *J* = 8.7 Hz, 2H, Har), 7.16 (d, *J* = 8.7 Hz, 2H, Har). **HPLC** (Chiralpak AD-H, *n*-hexane/2-propanol = 70/30, 1.0 mL/min, λ = 254 nm); t_R = 11.5 min (minor, *R*), 41.8 min (major, *S*).

(*S*)-Ethyl 1-((*R*)-2-nitro-1-phenylethyl)-2-oxocyclopentanecarboxylate (5aa).[5] Colorless solid. **^{1}H NMR** (500 MHz, $CDCl_3$) δ 1.27 (t, *J* = 7.2 Hz, 3H, CH_3), 1.80–2.09 (m, 4H, CH_2), 2.36 (m, 2H, CH_2CO), 4.07 (dd, *J* = 10.9, 3.8 Hz, 1H, CHPh), 4.21 (q, *J* = 7.1 Hz, 1H, $CHHCH_3$), 4.22 (q, *J* = 7.1 Hz, 1H, $CHHCH_3$), 5.01 (dd, *J* = 13.5, 11.0 Hz, 1H, $CHHNO_2$), 5.17 (dd, *J* = 13.5, 3.7 Hz, 1H, $CHHNO_2$), 7.20–7.35 (m, 5H, Har). **HPLC** (Chiralcel OD, *n*-hexane/2-propanol = 80/20, 1.0 mL/min, λ = 220 nm); t_R (major diastereoisomer) = 9.2 min (major), 12.4 min (minor).

(*S*)-Ethyl 1-((*R*)-2-nitro-1-phenylethyl)-2-oxocyclohexanecarboxylate (5ab).[5] Colorless oil. **^{1}H NMR** (500 MHz, $CDCl_3$) δ 1.26 (t, *J* = 7.1 Hz, 3H, CH_3), 1.44–1.74 (m, 4H, CH_2), 2.00–2.12 (m, 2H, CH_2), 2.42–2.54 (m, 2H, CH_2CO), 4.00 (dd, *J* = 11.3, 3.2 Hz, 1H, CH), 4.20 (m, 2H, OCH_2), 4.79 (dd, *J* = 13.5, 11.4 Hz, 1H, $CHHNO_2$), 5.06 (dd, *J* = 13.5, 3.3 Hz, 1H, $CHHNO_2$), 7.14–7.17 (m, 2H, Har), 7.25–7.30 (m, 3H, Har). **HPLC** (Chiralcel OD, *n*-hexane/2-propanol = 95/5, 1.0 mL/min, λ = 220 nm); t_R (major diastereoisomer) = 121.8 min (major), 15.4 min (minor).

(*S*)-Methyl 1-((*R*)-2-nitro-1-phenylethyl)-2-oxocycloheptanecarboxylate (5ac).[4] Colorless solid. **^{1}H NMR** (500 MHz, $CDCl_3$) δ 1.40–1.94 (m, 8H, CH_2), 2.50–2.63 (m, 2H, CH_2CO), 3.77 (s, 3H, CH_3O), 4.06 (dd, *J* = 10.0, 4.2 Hz, 1H, CH), 4.92 (dd, *J* = 13.6, 10.0 Hz, 1H, $CHHNO_2$), 4.96 (dd, *J* = 13.6, 4.2 Hz, 1H, $CHHNO_2$), 7.14–7.21 (m, 2H, Har), 7.28–7.34 (m, 3H, Har). **HPLC** (Chiralcel OD, *n*-hexane/2-propanol = 95/5, 1.0 mL/min, λ = 220 nm); t_R (major diastereoisomer) = 14.6 min (major), 30.5 min (minor).

(*R*)-2-Acetyl-2-((*R*)-2-nitro-1-phenylethyl)cyclopentanone (5ad).[5] Colorless solid. Major diastereoisomer. **^{1}H NMR** (500 MHz, $CDCl_3$) δ 1.72 (m, 3H), 1.98 (m, 1H), 2.19 (m, 1H), 2.32 (s, 3H, CH_3), 2.57 (m, 1H, CH), 4.39 (dd, *J* = 11.5, 3.9 Hz, 1H, CHPh),

4.51 (dd, *J* = 13.6, 3.9 Hz, 1H, $C\underline{H}HNO_2$), 4.86 (dd, *J* = 13.6, 11.5 Hz, 1H, $CH\underline{H}NO_2$), 7.24-7.33 (m, 5H, $\underline{H}$ar). **HPLC** (Chiralcel OD, *n*-hexane/2-propanol = 70/30, 1.0 mL/min, λ= 220 nm); t_R (major diastereoisomer) = 14.3 min (major), 44.9 min (minor).

(*R*)-3-acetyl-3-((*R*)-2-nitro-1-phenylethyl)dihydrofuran-2(3H)-one (5ae).[5] Colorless solid. Major diastereoisomer. **^{1}H NMR** (500 MHz, $CDCl_3$) δ 2.23–2.32 (m, 1H), 2.49 (s, 3H, $C\underline{H}_3$), 2.84 (ddd, *J* = 13.2, 7.8, 4.2 Hz, 1H), 3.85 (td, *J* = 8.9, 4.2 Hz, 1H, $C\underline{H}HO$), 4.03 (dt, *J* = 8.9, 7.8 Hz, 1H, $CH\underline{H}O$), 4.49–4.56 (m, 2H, $C\underline{H}HNO_2$ and $C\underline{H}Ph$), 4.85 (dd, *J* = 12.6, 10.3 Hz, 1H, $CH\underline{H}NO_2$), 7.32–7.39 (m, 5H). **HPLC** (Chiralcel OD, *n*-hexane/2-propanol = 70/30, 1.0 mL/min, λ = 220 nm); t_R (major diastereoisomer) = 25.1 min (major), 54.5 min (minor).

(*S*)-Ethyl 1-((*R*)1-(4-chlorophenyl)-2-nitroethyl)-2-oxocyclopentanecarboxylate (5ba).[6] Colorless oil. **^{1}H NMR** (500 MHz, $CDCl_3$) 1.26 (t, 3H, *J* = 7.1 Hz, $C\underline{H}_3$), 2.11-1.82 (m, 4H, $C\underline{H}_2$), 2.38 (m, 2H, $C\underline{H}_2CO$), 4.03 (dd, *J* = 11.1, 3.7 Hz, 1H, $C\underline{H}$), 4.20 (q, 1H, *J* = 7.1 Hz, $OC\underline{H}H$), 4.21 (q, 1H, *J* = 7.1 Hz, $OCH\underline{H}$), 4.97 (dd, *J* = 13.7, 11.1 Hz, 1H, $C\underline{H}HNO_2$), 5.15 (dd, 1H, *J* = 13.7, 3.7 Hz, $CH\underline{H}NO_2$), 7.22 (d, 2H, *J* = 8.6 Hz, Har), 7.28 (d, 2H, *J* = 8.6 Hz, Har). **HPLC** (Chiralcel OD column, *n*-hexane/2-propanol = 90/10, 1.0 mL/min, λ = 220 nm); t_R (major diastereoisomer) = 15.1 min (major), 24.0 min (minor).

(*S*)-Ethyl 1-((*R*)1-(4-fluorophenyl)-2-nitroethyl)-2-oxocyclopentanecarboxylate (5ca).[6] Colorless oil. **^{1}H NMR** (500 MHz, $CDCl_3$) δ 1.26 (t, *J* = 7.2 Hz, 3H, $C\underline{H}_3$), 1.81-2.11 (m, 4H, $C\underline{H}_2$), 2.38 (m, 2H, $C\underline{H}_2CO$), 4.04 (dd, *J* = 11.2, 3.7 Hz, 1H, $C\underline{H}$), 4.19 (q, *J* =7.2 Hz, 1H, $OC\underline{H}_2$), 4.20 (q, *J* =7.2 Hz, 1H, $OC\underline{H}_2$), 4.96 (dd, *J* = 13,5, 11.1 Hz, 1H, $CH\underline{H}NO_2$), 5.14 (dd, *J* = 13.5, 3.8 Hz, 1H, $C\underline{H}HNO_2$), 7.00 (m, 2H, $\underline{H}$ar), 7.26 (m, 2H, $\underline{H}$ar). **HPLC** (Chiralcel OD, *n*-hexane/2-propanol = 90/10, 0.5 mL/min, λ = 220 nm); t_R (major diastereoisomer) = 24.2 min (major), 40.6 min (minor).

(*S*)-Ethyl 1-((*R*)1-(4-methoxyphenyl)-2-nitroethyl)-2-oxocyclopentanecarboxylate (5da).[6] Colorless oil. **^{1}H NMR** (500 MHz, $CDCl_3$) δ 1.27 (t, *J* = 7.1 Hz, 3H, $C\underline{H}_3$), 1.78-2.05 (m, 4H, $C\underline{H}_2$), 2.35 (m, 2H, $C\underline{H}_2CO$), 3.77 (s, 3H, $OC\underline{H}_3$), 4.05 (dd, *J* =11.1, 3.8 Hz, 1H, $C\underline{H}$), 4.20 (q, *J* = 7.2 Hz, 2H, OCH_2), 4.96 (dd, *J* =13.4, 11.1 Hz, 1H, $C\underline{H}HNO_2$), 5.12 (dd, *J* = 13.4, 3.8 Hz, 1H, $CH\underline{H}NO_2$), 6.82 (d, *J* = 8.8 Hz, 2H, $\underline{H}$ar), 7.18 (d, *J* = 8.8 Hz, 2H, $\underline{H}$ar). **HPLC** (Chiralpak AD-H column, *n*-hexane/2-propanol =

97/3, 1.0 mL/min, λ = 210 nm); t_R (major diastereoisomer) = 27.5 min (minor), 33.1 min (major).

(*S*)-2-Nitro-2-((*S*)-2-nitro-1-phenylethyl)cyclohexanone (7aa).[7] Colorless solid. **^{1}H NMR** (500 MHz, $CDCl_3$) δ 1.53-1.80 (m, 4H, CH_2), 2.05 (m, 1H, CHHCO), 2.30 (ddd, *J* = 14.9, 6.3, 3.4 Hz, 1H, CHHCO), 2.58 (m, 1H, $CHHCNO_2$), 2.66 (dtd, *J* = 13.4, 4.2, 1.5 Hz, 1H, $CHHCNO_2$), 4.30 (dd, *J* = 11.1, 3.1 Hz, 1H, CHPh), 4.70 (dd, *J* = 13.8, 11.1 Hz, 1H, $CHHNO_2$), 5.14 (dd, *J* = 13.7, 3.2 Hz, 1H, $CHHNO_2$), 7.06-7.09 (m, 2H, Har), 7.32-7.37 (m, 3H, Har). **HPLC** (Chiralcel OD, *n*-hexane/2-propanol = 90:10, 1.0 mL/min, λ= 220 nm); t_R = 22.4 min (major), 38.8 min (minor).

(*S*)-2-((*S*)-1-(4-Chlorophenyl)-2-nitroethyl)-2-nitrocyclohexanone (7ba). [7] Yellow oil. **^{1}H NMR** (500 MHz, $CDCl_3$) δ 1.57 (m, 1H), 1.64-1.81 (m, 3H), 2.05 (m, 1H), 2.31 (dq, *J* = 15.0, 3.5 Hz, 1H), 2.57 (m, 1H), 2.65 (m, 1H), 4.27 (dd, *J* = 11.1, 3.2 Hz, 1H, CHAr), 4.63 (dd, *J* = 13.8, 11.1 Hz, 1H, $CHHNO_2$), 5.10 (dd, *J* = 13.8, 3.2 Hz, 1H, $CHHNO_2$), 7.04 (d, *J* = 8.5 Hz, 2H, Har), 7.31 (d, *J* = 8.5 Hz, 2H, Har**). HPLC** (Chiralcel OD, *n*-hexane/2-propanol = 90:10, 1.0 mL/min, λ = 220 nm); t_R = 22.9 min (major), 48.8 min (minor).

(*S*)-2-[(*S*)-1-(4-Methoxyphenyl)-2-nitroethyl)-2-nitrocyclohexanone (7da). [7] Yellow oil. **^{1}H NMR** (500 MHz, $CDCl_3$) δ 1.58 (m, 1H), 1.64-1.81 (m, 4H, CH_2), 2.04 (m, 1H), 2.33 (dq, *J* = 14.8, 3.5 Hz, 1H), 2.57 (ddd, *J* = 13.5, 12.1, 5.8 Hz, 1H), 2.64 (dtd, *J* = 13.5, 4.3, 1.5 Hz, 1H), 3.78 (s, 3H, CH_3), 4.24 (dd, *J* = 11.1, 3.2 Hz, 1H, CHAr), 4.66 (dd, *J* = 13.5, 11.1 Hz, 1H, $CHHNO_2$), 5.09 (dd, *J* = 13.6, 3.2 Hz, 1H, $CHHNO_2$), 6.84 (d, *J* = 8.8 Hz, 2H, Har), 7.00 (d, *J* = 8.8 Hz, 2H, Har). **HPLC** (Chiralcel OD, *n*-hexane/2-propanol = 90:10, 1.0 mL/min, λ = 220 nm); t_R = 18.1 min (major), 30.3 min (minor).

(2*S*,3*S*)-Ethyl 2-Methyl-2,4-dinitro-3-phenylbutanoate (7ab).[8] Colorless solid. **^{1}H NMR** (500 MHz, $CDCl_3$) δ 1.31 (t, *J* = 7.2 Hz, 3H, CH_3CH_2); 1.64 (s, 3H, CH_3); 4.32 (q, *J* = 7.2 Hz, 3H, CH_2CH_3); 4.40 (dd, *J* = 10.4, 3.1 Hz, 1H, CHPh); 5.06 (dd, *J* = 14.0, 10.4 Hz, 1H, $CHHNO_2$); 5.12 (dd, *J* = 14.0, 3.2 Hz, 1H, $CHHNO_2$); 7.12–7.14 (m, 2H, Har); 7.34-7.36 (m, 3H, Har). **HPLC** (Chiralcel OD, *n*-hexane/2-propanol = 80:20, 1.0 mL/min, λ = 220 nm); *anti*-adduct: t_R = 12.3 min (major), 28.8 min (major) (er 74:26); *syn*-adduct: t_R = 14.1 min (major), 16.6 min (minor) (er 60:40).

2. NMR spectra for new compounds

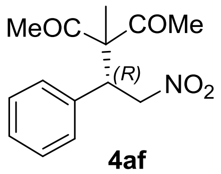

4af

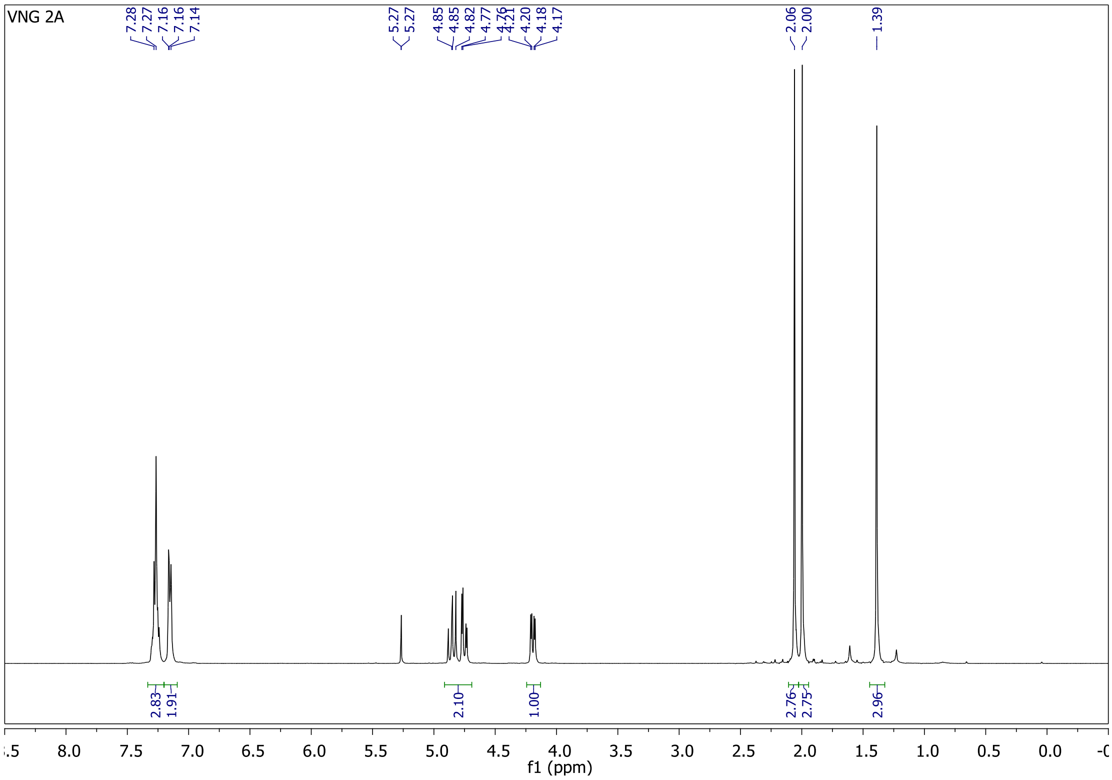

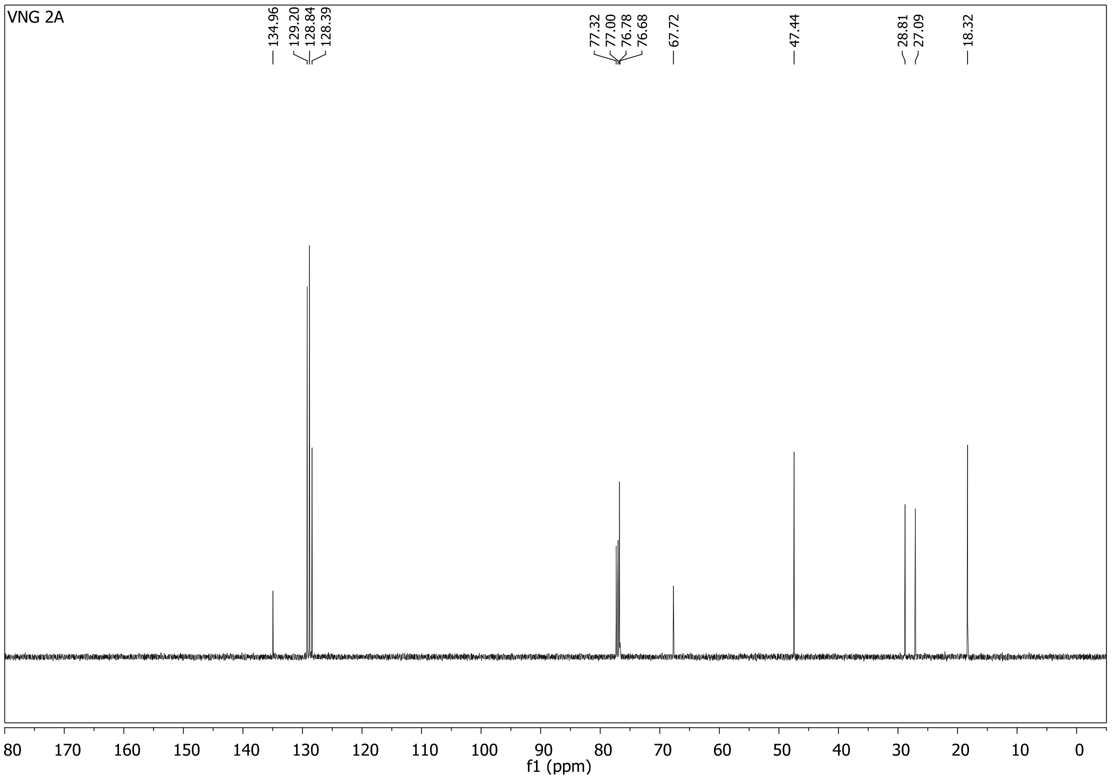

3. HPLC Profiles of the Nitro-Michael Products.

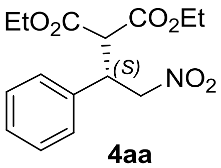

4aa

Racemic molecule:

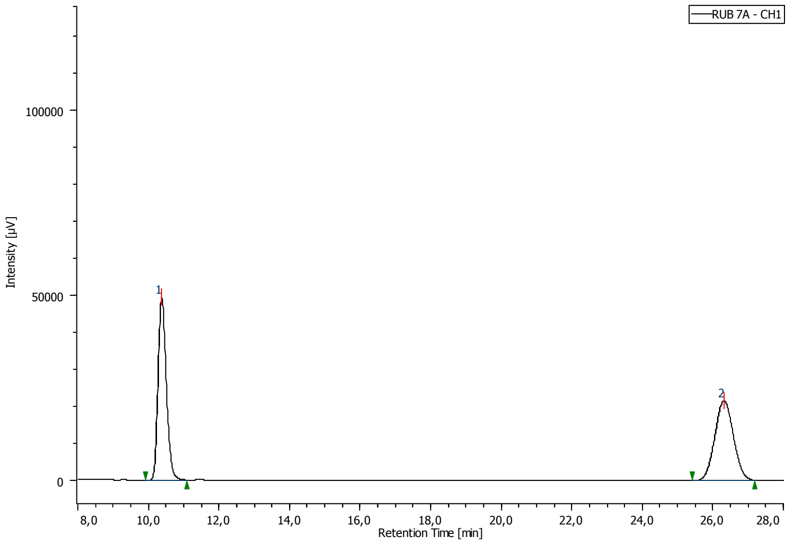

#	t_R	Area	Height	Area%	Height%	Symmetry Factor
1	10,375	799837	49174	50,348	69,552	1,237
2	26,292	788792	21527	49,652	30,448	1,034

HPLC profile for entry 5, table 1. 90:10 er.

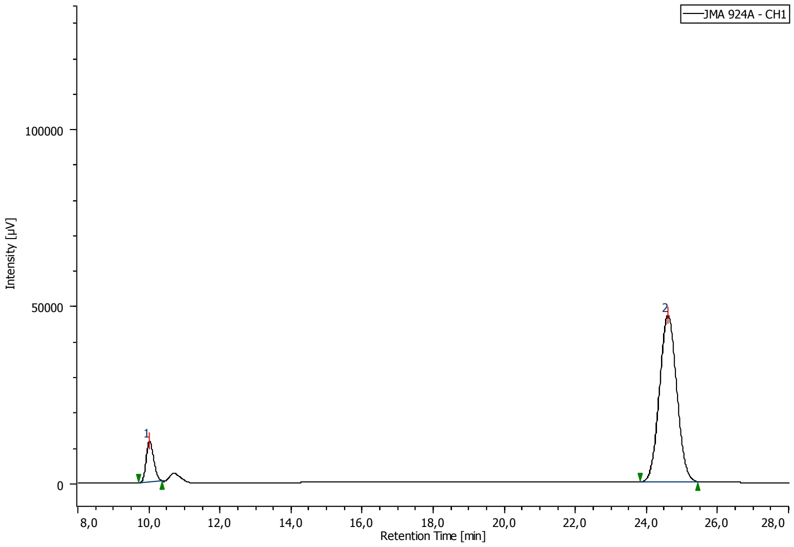

#	tR	Area	Height	Area%	Height%	Symmetry Factor
1	10,017	172995	11618	9,844	19,852	1,189
2	24,583	1584300	46904	90,156	80,148	1,044

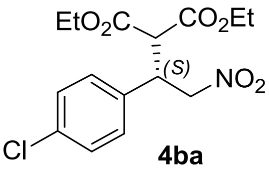

4ba

Racemic molecule:

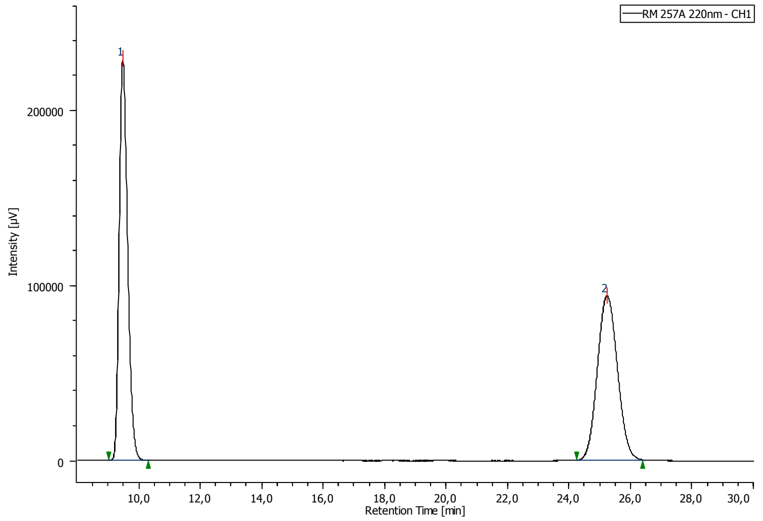

#	tR	Area	Height	Area%	Height%	Symmetry Factor
1	9,483	4269299	228674	50,230	70,937	1,279
2	25,217	4230130	93688	49,770	29,063	1,073

HPLC profile for entry 1, table 2. 83:17 er.

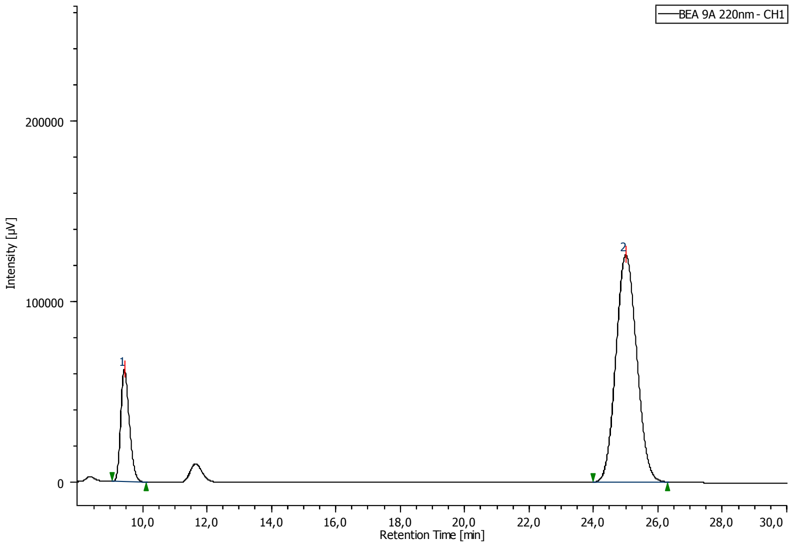

#	tR	Area	Height	Area%	Height%	Symmetry Factor
1	9,442	1148279	62097	16,903	33,086	1,254
2	24,983	5645227	125586	83,097	66,914	1,081

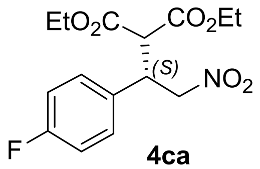

4ca

Racemic molecule:

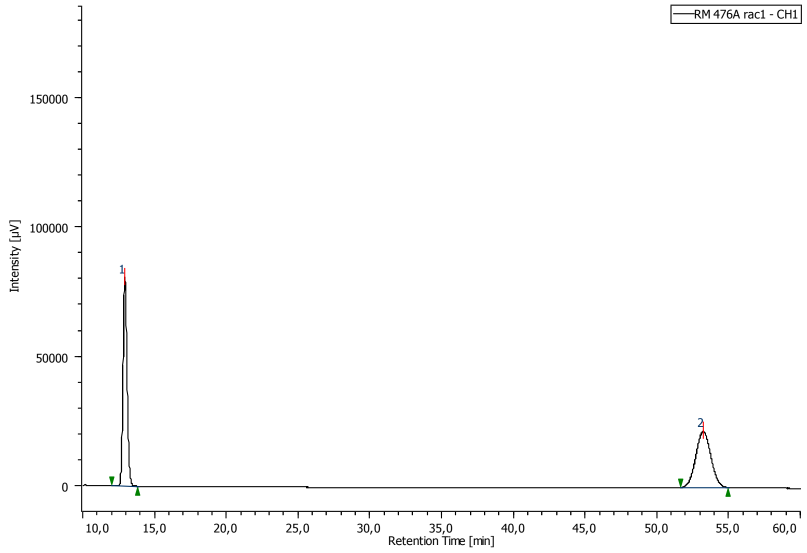

#	tR	Area	Height	Area%	Height%	Symmetry Factor
1	12,933	1590400	80477	50,138	78,766	1,163
2	53,175	1581672	21695	49,862	21,234	1,037

HPLC profile for entry 2, table 2. 86:14 er.

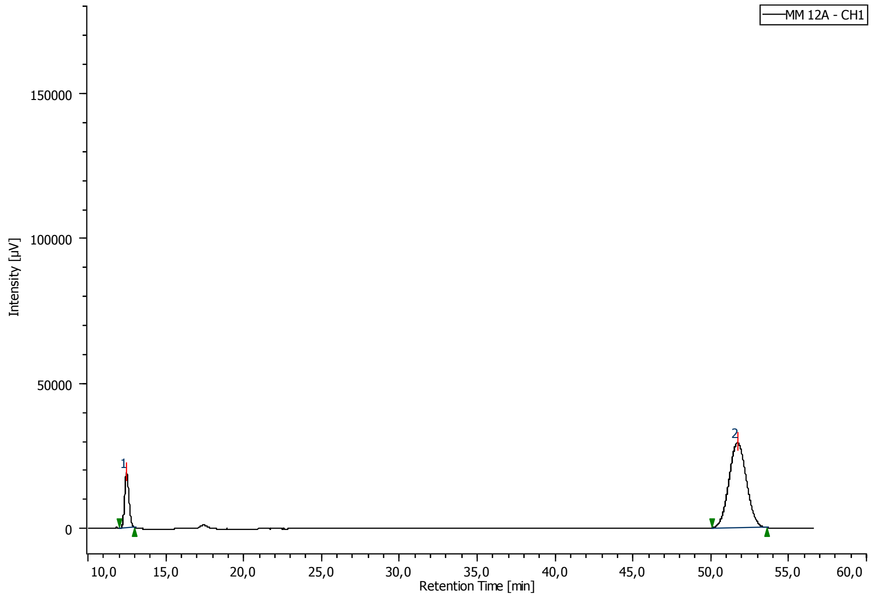

#	tR	Area	Height	Area%	Height%	Symmetry Factor
1	12,483	367409	18932	13,921	39,276	1,174
2	51,658	2271851	29271	86,079	60,724	1,056

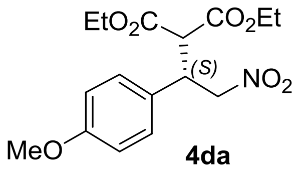

4da

Racemic molecule:

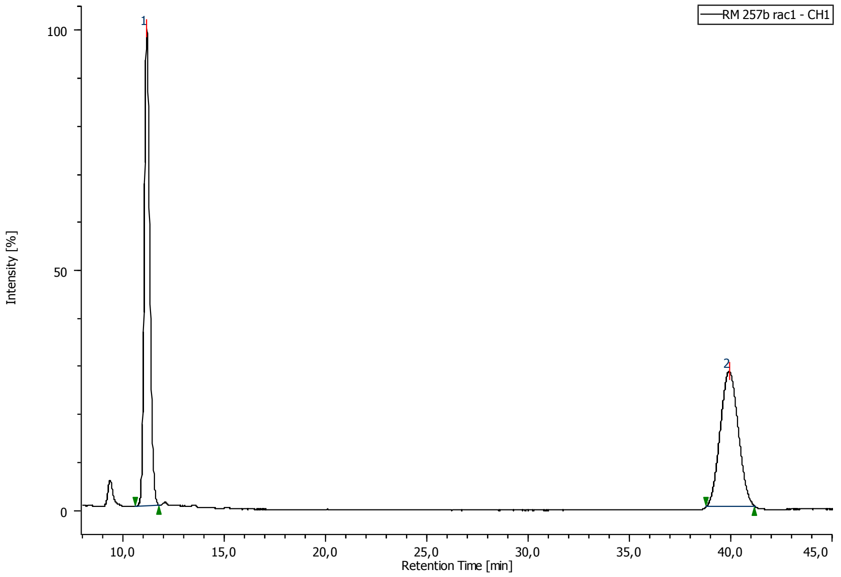

#	tR	Area	Height	Area%	Height%	Symmetry Factor
1	11,175	214500	11405	51,758	77,903	1,182
2	39,875	199931	3235	48,242	22,097	1,048

HPLC profile for entry 3, table 2. 89:11 er.

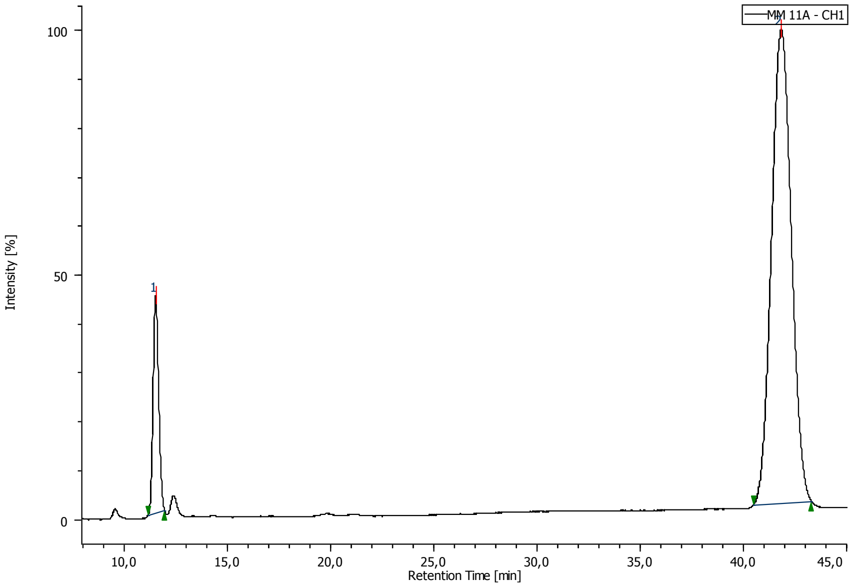

#	tR	Area	Height	Area%	Height%	Symmetry Factor
1	11,525	65493	3471	11,457	31,464	1,120
2	41,783	506173	7560	88,543	68,536	1,051

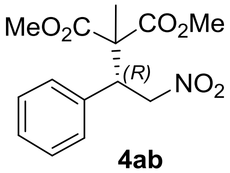

4ab

Racemic molecule:

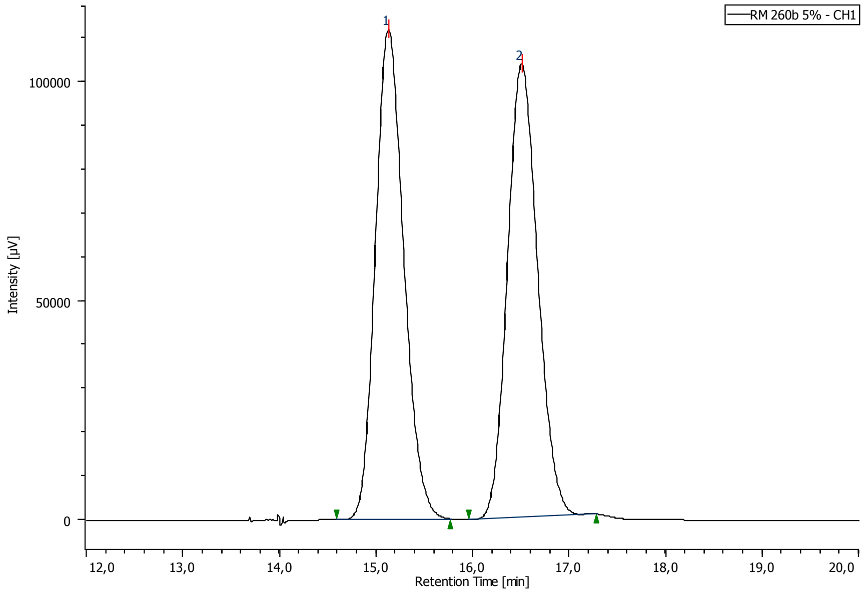

#	tR	Area	Height	Area%	Height%	Symmetry Factor
1	15,125	2283252	111593	50,339	51,938	1,178
2	16,508	2252481	103267	49,661	48,062	1,115

HPLC profile for entry 4, table 2. 92:8 er.

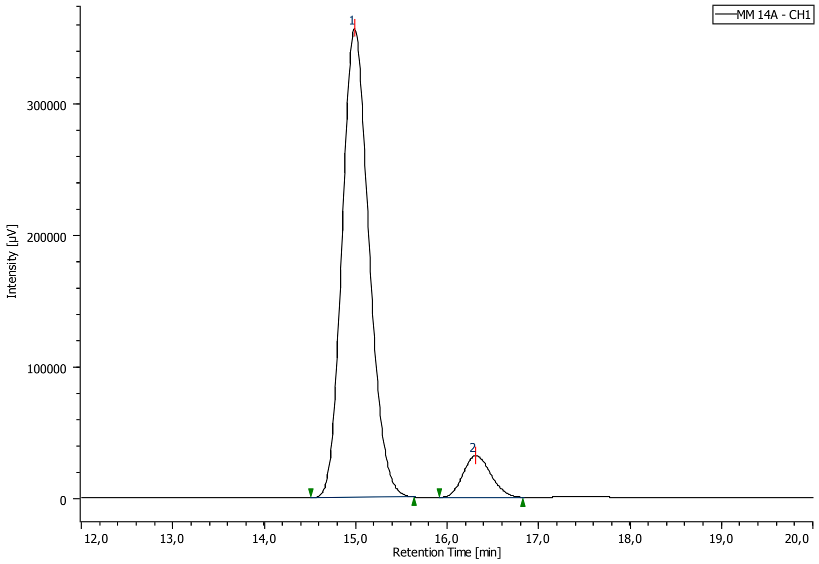

#	tR	Area	Height	Area%	Height%	Symmetry Factor
1	14,983	7273303	355446	91,545	91,786	1,165
2	16,308	671768	31807	8,455	8,214	1,152

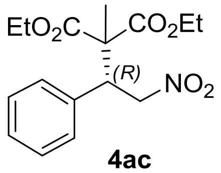

4ac

Racemic molecule:

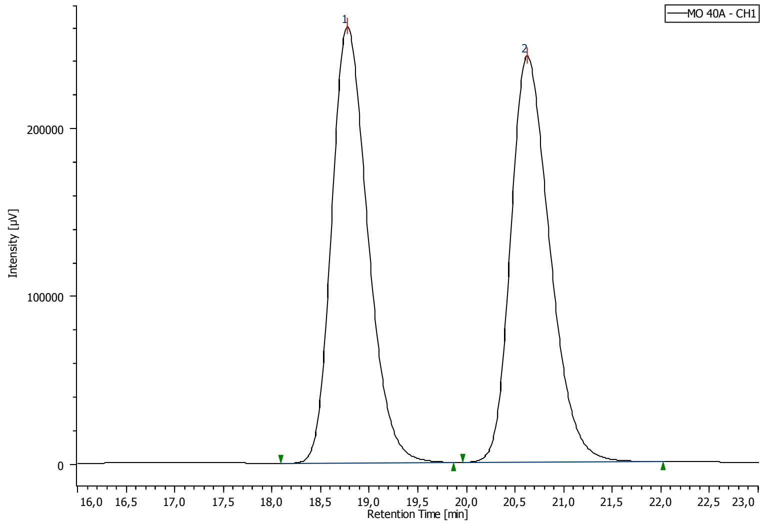

#	tR	Area	Height	Area%	Height%	Symmetry Factor
1	18,775	6686021	259376	49,801	51,761	1,245
2	20,617	6739497	241725	50,199	48,239	1,305

HPLC profile for entry 10, table 2. 91:9 er.

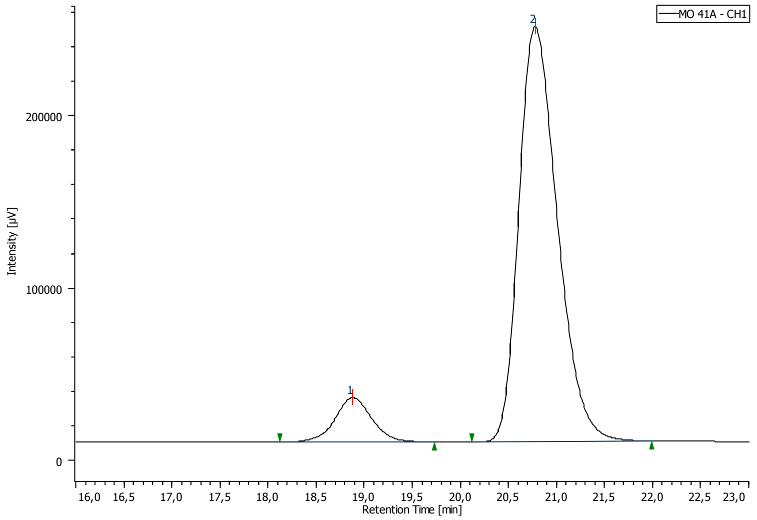

#	tR	Area	Height	Area%	Height%	Symmetry Factor
1	18,875	690331	25772	9,335	9,670	1,027
2	20,775	6704599	240753	90,665	90,330	1,300

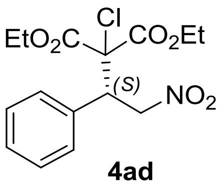

4ad

Racemic molecule:

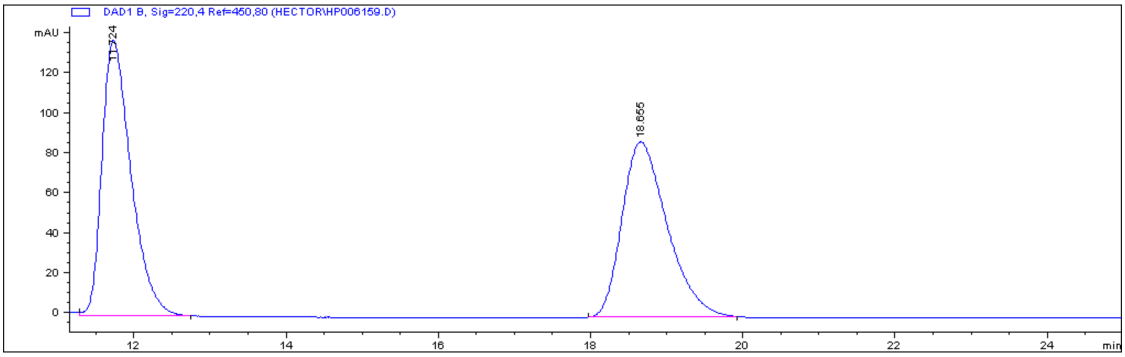

#	Time	Area	Height	Width	Area%	Symmetry
1	11.724	3679.4	138.3	0.4	50.253	0.604
2	18.655	3642.3	87.8	0.6025	49.747	0.676

HPLC profile for entry 5, table 2. 92:8 er.

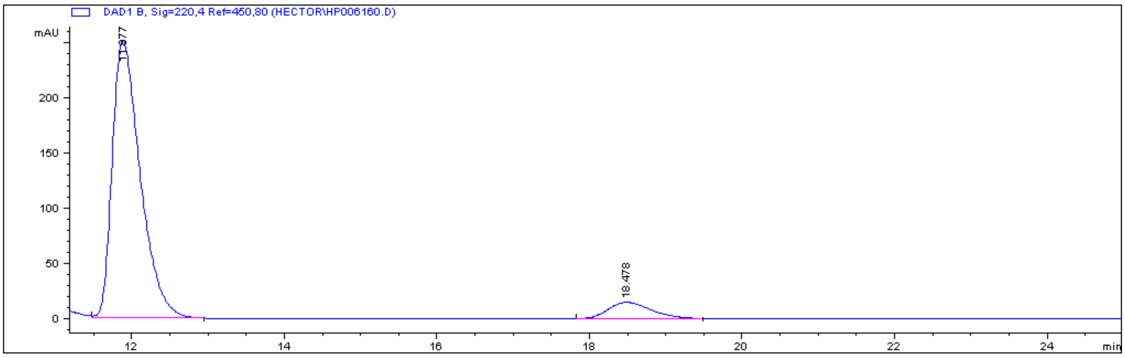

#	Time	Area	Height	Width	Area%	Symmetry
1	11.877	6450.4	252.6	0.3875	91.717	0.563
2	18.478	582.5	14.8	0.5039	8.283	0.674

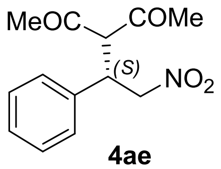

4ae

Racemic molecule:

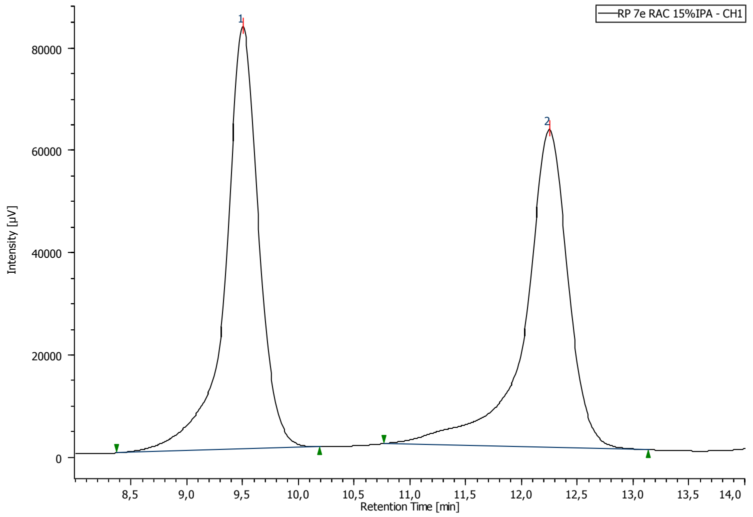

#	tR	Area	Height	Area%	Height%	Symmetry Factor
1	9,500	1719557	82405	50,406	57,039	0,826
2	12,242	1691837	62066	49,594	42,961	0,724

HPLC profile for entry 12, table 2. 92:8 er.

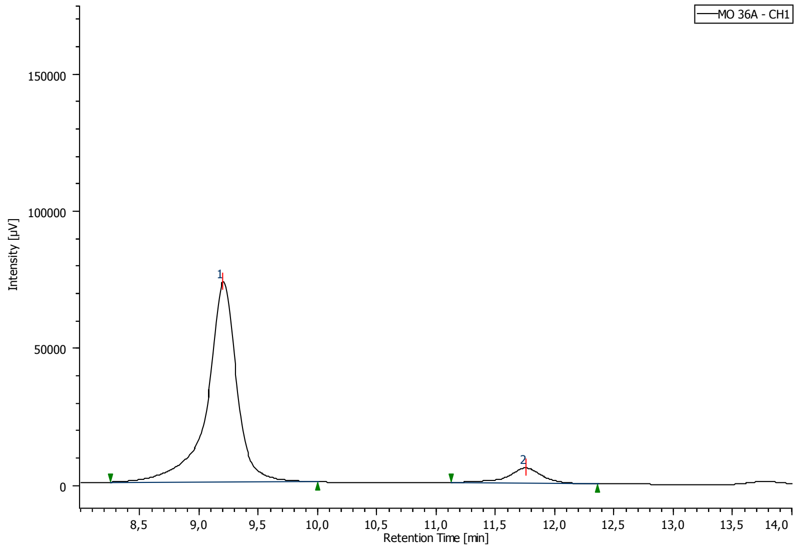

#	tR	Area	Height	Area%	Height%	Symmetry Factor
1	9,200	1262224	72827	91,684	92,909	0,817
2	11,750	114483	5559	8,316	7,091	0,875

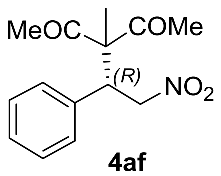

4af

Racemic molecule:

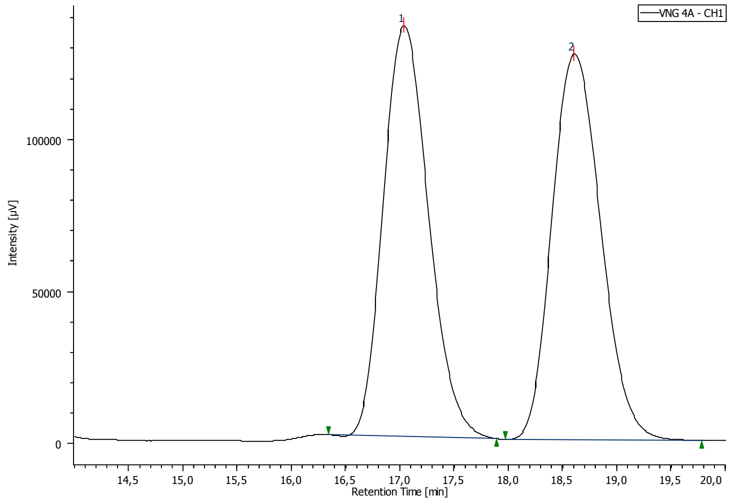

#	tR	Area	Height	Area%	Height%	Symmetry Factor
1	17,033	3902459	134727	49,656	51,545	1,196
2	18,600	3956485	126651	50,344	48,455	1,180

HPLC profile for entry 13, table 2. 93:7 er.

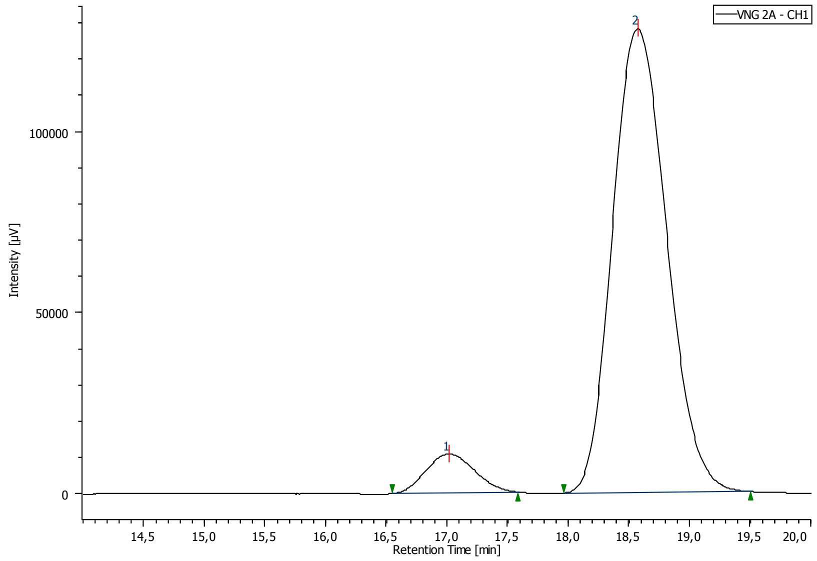

#	tR	Area	Height	Area%	Height%	Symmetry Factor
1	17,017	299487	10738	7,024	7,754	1,104
2	18,567	3964206	127736	92,976	92,246	1,161

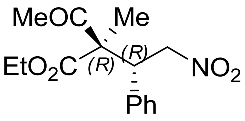

4ag

Racemic molecule:

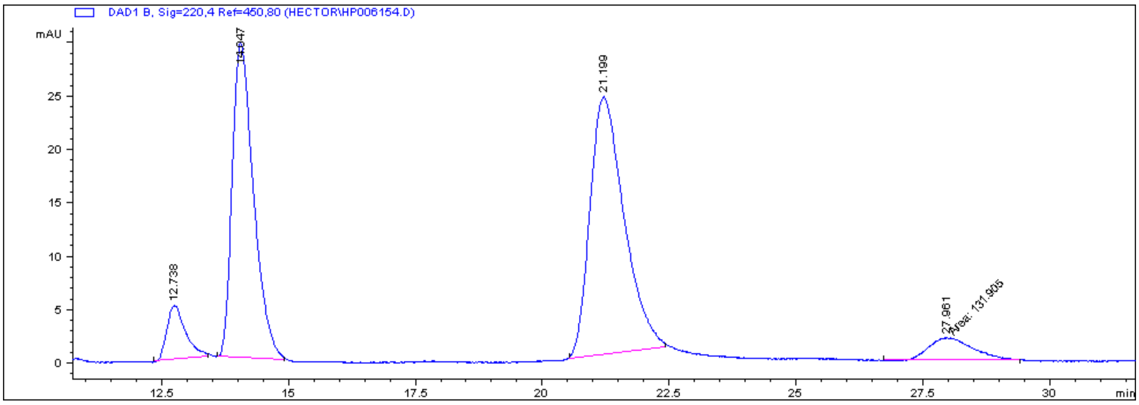

#	Time	Area	Height	Width	Area%	Symmetry
1	12.738	131.2	5.1	0.3114	5.804	0.606
2	14.047	985.1	29.5	0.4216	43.582	0.597
3	21.199	1012.1	24.2	0.5858	44.777	0.63
4	27.961	131.9	2.1	1.0383	5.836	0.532

HPLC profile for entry 8, table 2. 75/25 dr; 92:8 er.

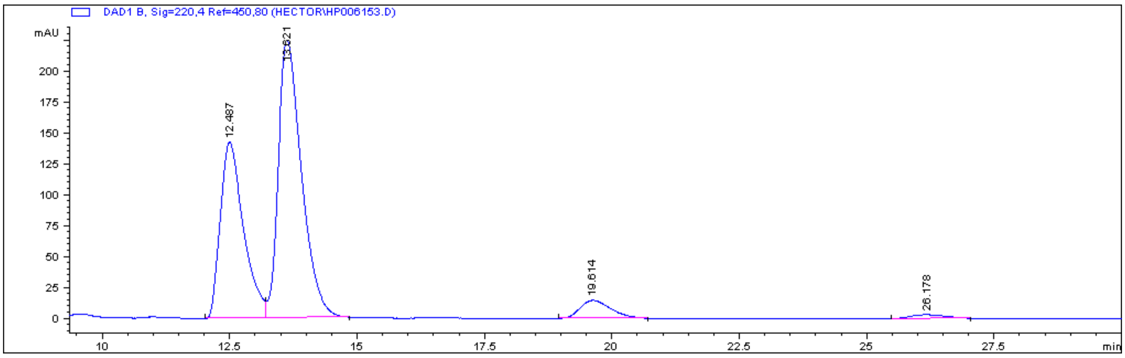

#	Time	Area	Height	Width	Area%	Symmetry
1	12.487	4519.2	142.4	0.4663	36.346	0.605
2	13.621	7140.1	224.7	0.4827	57.425	0.584
3	19.614	622.2	14.7	0.5191	5.004	0.679
4	26.178	152.4	3.2	0.5748	1.226	0.832

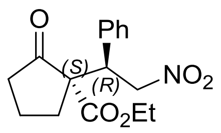

5aa

Racemic molecule:

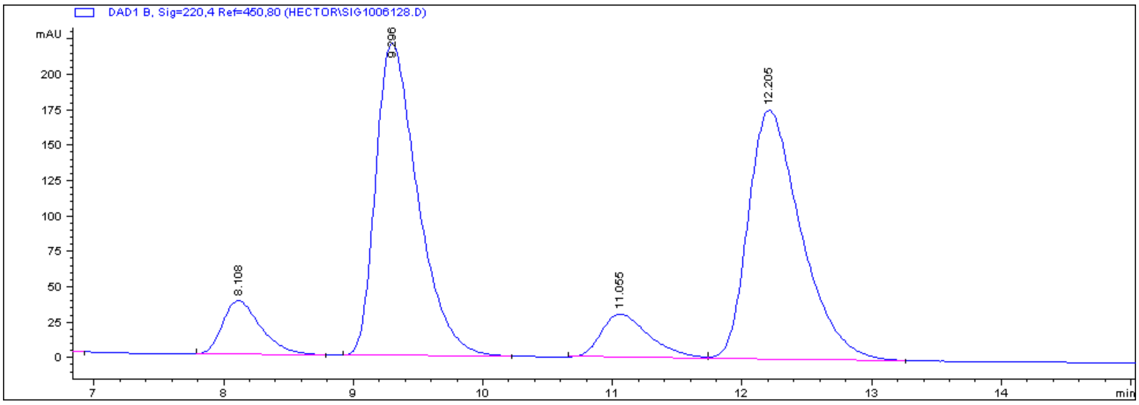

#	Time	Area	Height	Width	Area%	Symmetry
1	8.108	772.2	37.9	0.3146	6.702	0.642
2	9.296	4974.2	220.7	0.3462	43.173	0.604
3	11.055	790.4	30.6	0.3652	6.860	0.613
4	12.205	4984.9	175.9	0.433	43.265	0.625

HPLC profile for entry 15, table 1. 89/11 dr; 95:5 er.

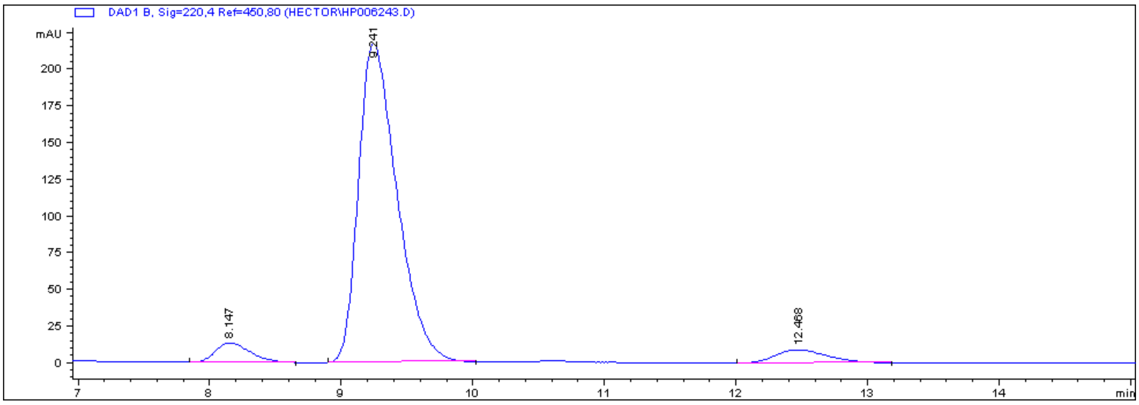

#	Time	Area	Height	Width	Area%	Symmetry
1	8.147	243.5	13.3	0.2655	4.950	0.659
2	9.241	4427.6	217.5	0.3111	90.025	0.597
3	12.468	247.1	9.1	0.3345	5.025	0.719

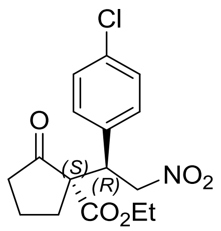

5ba

Racemic molecule:

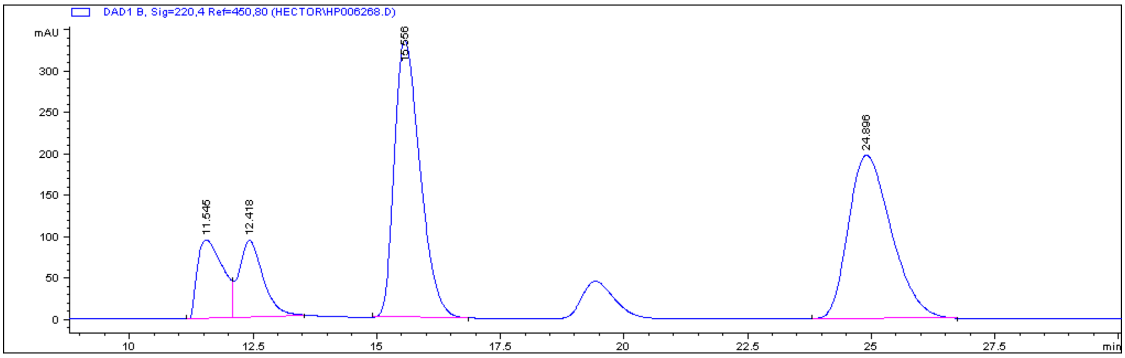

#	Time	Area	Height	Width	Area%	Symmetry
1	11.545	3443.7	94.7	0.5297	11.224	0.501
2	12.418	3123.4	93.1	0.4843	10.180	0.691
3	15.556	12156.1	335.4	0.554	39.620	0.637
4	24.896	11958.1	197.6	0.8563	38.975	0.698

HPLC profile for entry 2, table 3. 90/10 dr; 94:6 er.

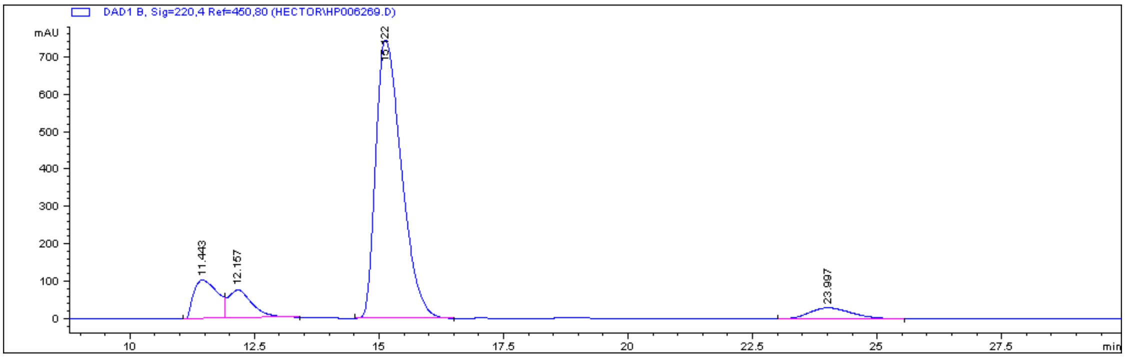

#	Time	Area	Height	Width	Area%	Symmetry
1	11.443	3506.3	103.3	0.5048	10.143	0.554
2	12.157	2560.8	76.1	0.4598	7.407	0.565
3	15.122	26758.7	744.1	0.554	77.404	0.592
4	23.997	1744.6	29.4	0.7112	5.046	0.741

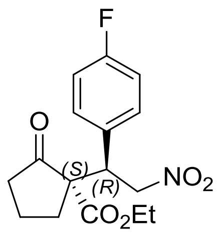

5ca

Racemic molecule:

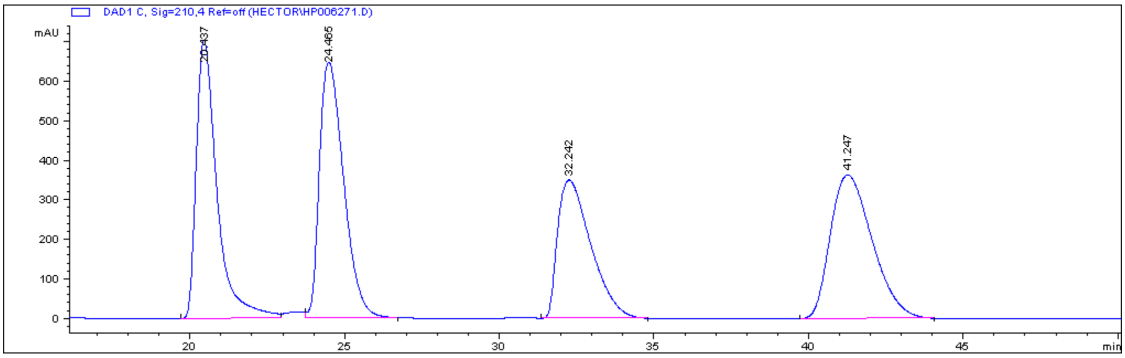

#	Time	Area	Height	Width	Area%	Symmetry
1	20.437	33057.3	704.9	0.6911	25.662	0.542
2	24.465	35332.8	648	0.7513	27.429	0.566
3	32.242	26142.1	349.7	0.8977	20.294	0.509
4	41.247	34284.6	364	1.1117	26.615	0.679

HPLC profile for entry 3, table 3. 89/11 dr; 93:7 er.

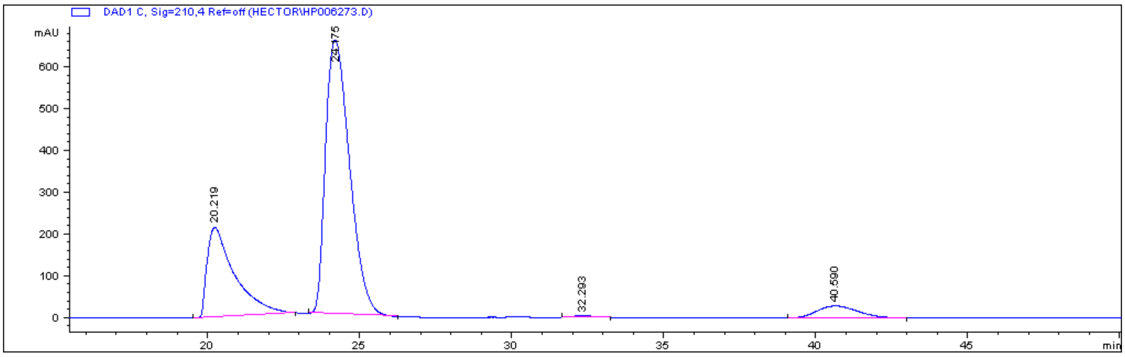

#	Time	Area	Height	Width	Area%	Symmetry
1	20.219	13530.1	215.3	0.8741	25.665	0.353
2	24.175	36262.5	656.3	0.7758	68.785	0.607
3	32.293	229.1	3.9	0.6973	0.435	0.777
4	40.59	2696.6	28.8	1.1065	5.115	0.705

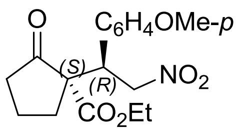

5da

Racemic molecule:

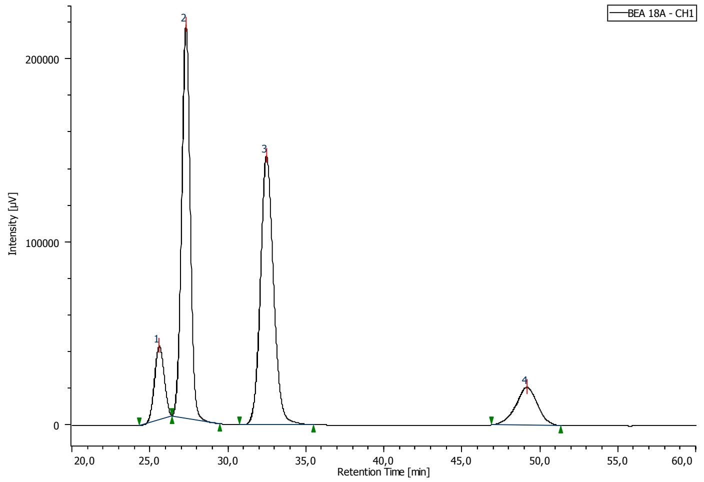

#	tR	Area	Height	Area%	Height%	Symmetry Factor
1	25,600	1708176	40011	8,412	9,501	1,010
2	27,317	8088582	214091	39,833	50,838	0,987
3	32,467	8517863	146421	41,947	34,769	1,114
4	49,117	1991760	20604	9,809	4,892	0,950

HPLC profile for entry 4, table 3. 89/11 dr; 95:5 er.

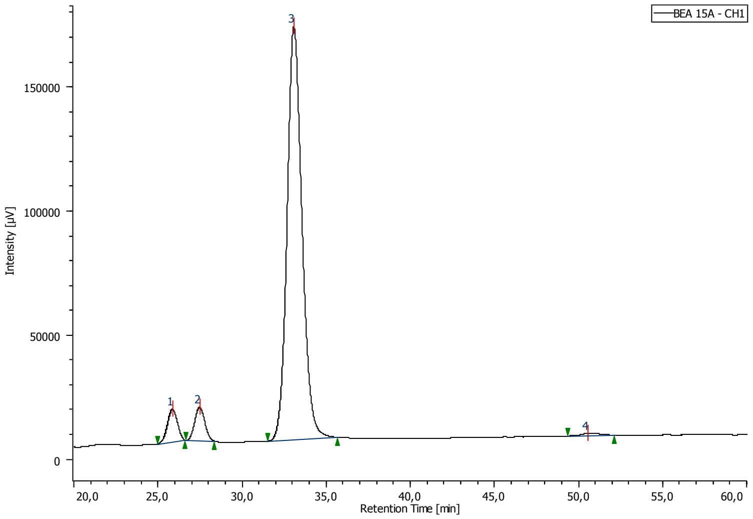

#	tR	Area	Height	Area%	Height%	Symmetry Factor
1	25,867	543032	13106	4,763	6,770	0,966
2	27,475	564460	13554	4,951	7,001	1,044
3	33,067	10218843	166097	89,634	85,794	1,146
4	50,517	74353	842	0,652	0,435	1,160

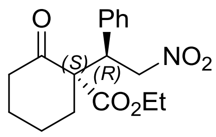

5ab

Racemic molecule:

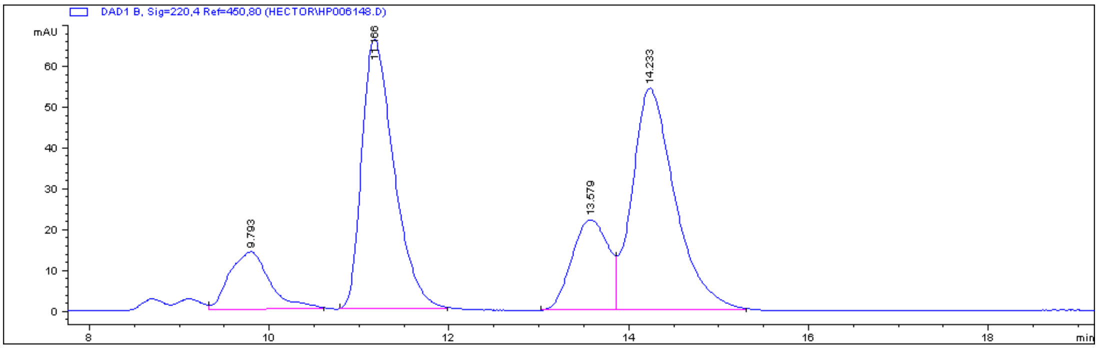

#	Time	Area	Height	Width	Area%	Symmetry
1	9.793	445.9	14.1	0.4162	9.876	1.033
2	11.166	1639.9	66	0.3794	36.317	0.607
3	13.579	629.4	22.2	0.3856	13.939	1.094
4	14.233	1800.2	54.3	0.47	39.868	0.675

HPLC profile for entry 6, table 3. 88/12 dr; 96:4 er.

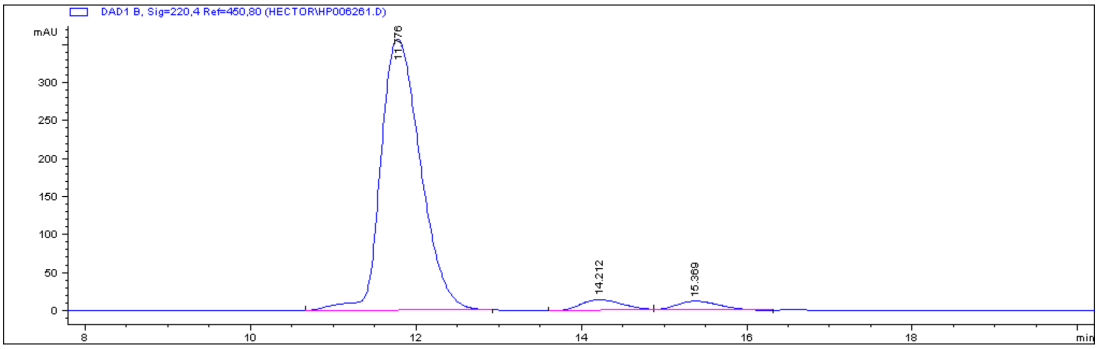

#	Time	Area	Height	Width	Area%	Symmetry
1	11.776	11928.8	357.3	0.5143	92.510	0.731
2	14.212	518	14.3	0.4324	4.017	0.742
3	15.369	447.8	12.1	0.4617	3.473	0.75

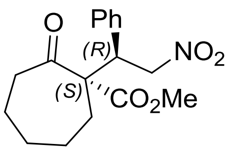

5ac

Racemic molecule:

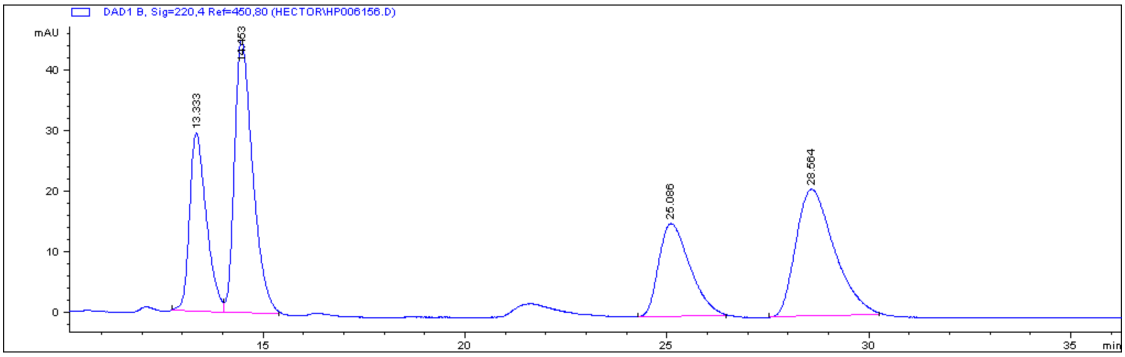

#	Time	Area	Height	Width	Area%	Symmetry
1	13.333	863.8	29.5	0.4183	19.187	0.638
2	14.453	1422.2	45.2	0.4537	31.589	0.603
3	25.086	827.2	15.4	0.6457	18.374	0.61
4	28.564	1388.9	21	0.7883	30.850	0.658

HPLC profile for entry 8, table 3. 82/18 dr; 96:4 er.

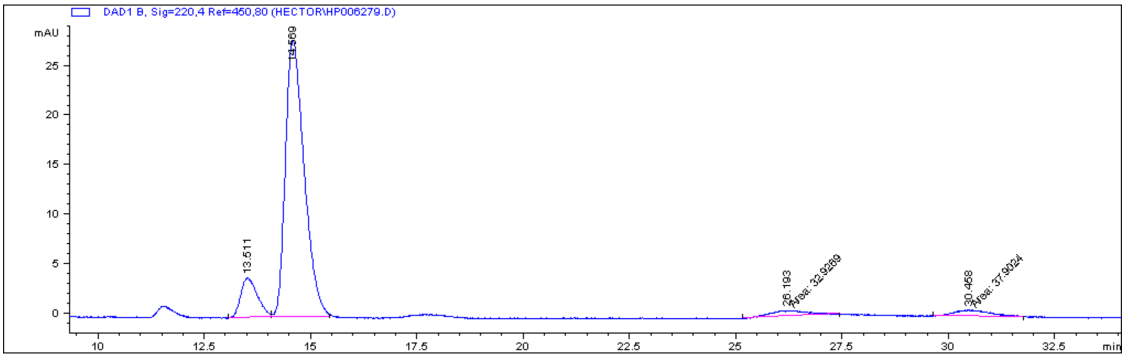

#	Time	Area	Height	Width	Area%	Symmetry
1	13.511	110.3	4	0.3349	10.591	0.675
2	14.569	860.3	28	0.4224	82.608	0.608
3	26.193	32.9	5.2E-1	1.0484	3.162	0.798
4	30.458	37.9	6.4E-1	0.9883	3.639	0.631

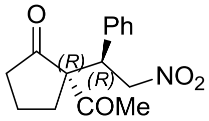

5ad

Racemic molecule:

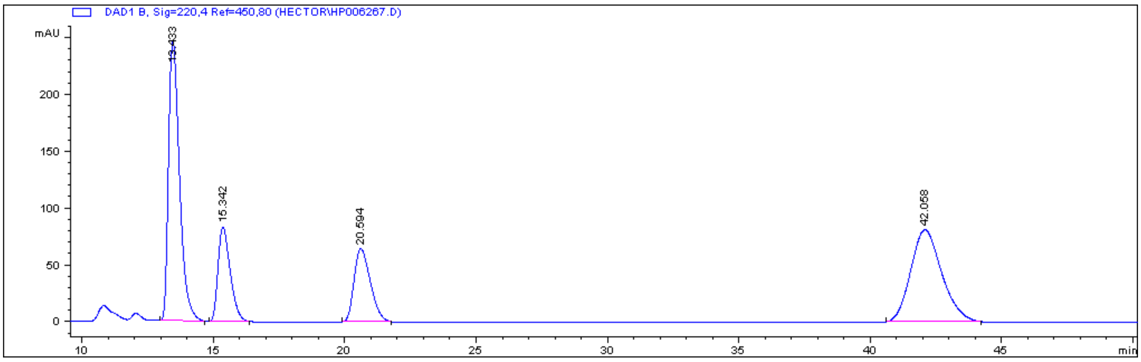

#	Time	Area	Height	Width	Area%	Symmetry
1	13.433	7077.5	246.8	0.437	36.660	0.586
2	15.342	2698.7	83.2	0.4841	13.979	0.634
3	20.594	2800.2	64.5	0.5928	14.505	0.682
4	42.058	6729.3	81.3	1.0067	34.856	0.793

HPLC profile for entry 9, table 3. 83/17 dr; 93:7 er.

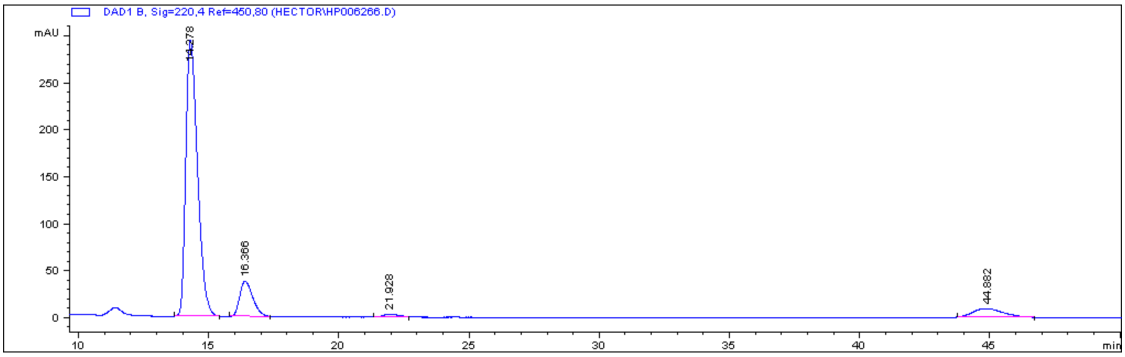

#	Time	Area	Height	Width	Area%	Symmetry
1	14.278	9284.7	294.3	0.4737	80.921	0.633
2	16.366	1320.2	37.8	0.4941	11.506	0.626
3	21.928	129.7	3.3	0.48	1.130	0.656
4	44.882	739.1	9.3	0.9665	6.442	0.806

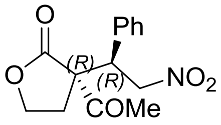

5ae

Racemic molecule:

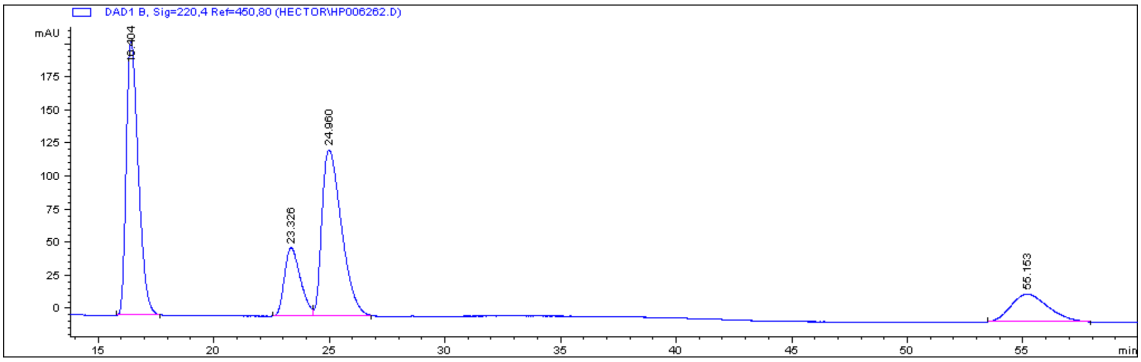

#	Time	Area	Height	Width	Area%	Symmetry
1	16.404	7547.3	207.8	0.5614	38.164	0.633
2	23.326	2482.5	51.6	0.5836	12.553	0.708
3	24.96	7411.7	125.6	0.8293	37.478	0.527
4	55.153	2334.5	20.8	1.3132	11.805	0.728

HPLC profile for entry 10, table 3. 70/30 dr; 92:8 er.

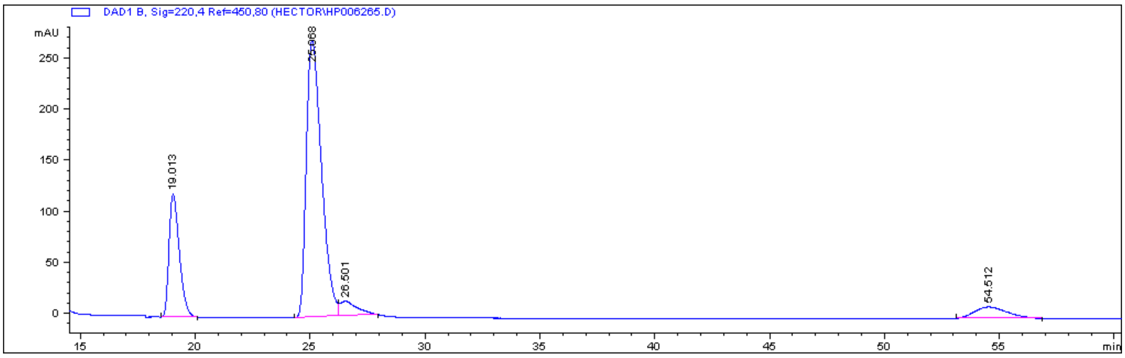

#	Time	Area	Height	Width	Area%	Symmetry
1	19.013	3765.9	120.4	0.4642	20.692	0.616
2	25.068	12527.7	271.3	0.6731	68.832	0.582
3	26.501	789.9	14.6	0.6471	4.340	0.37
4	54.512	1116.8	11.1	1.1891	6.136	0.721

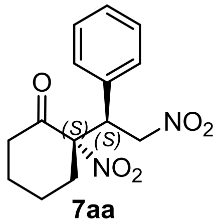

7aa

Racemic molecule:

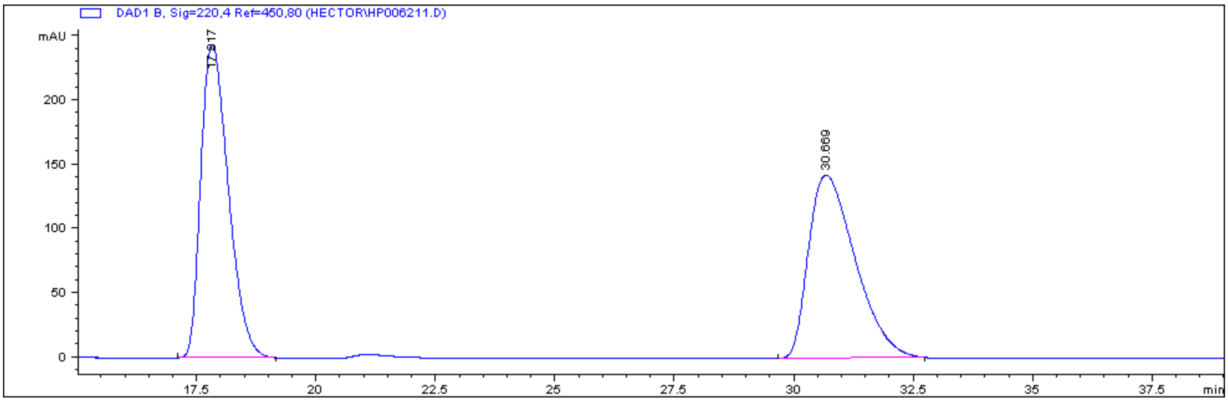

Peak Name	Time	Area	Height	Area%	Symmetry
1	17.817	9794.4	243.9	50.250	0.665
2	30.669	9697.1	142.6	49.750	0.57

HPLC profile for entry 6, table 4. 100:0 rd, 94:6 er.

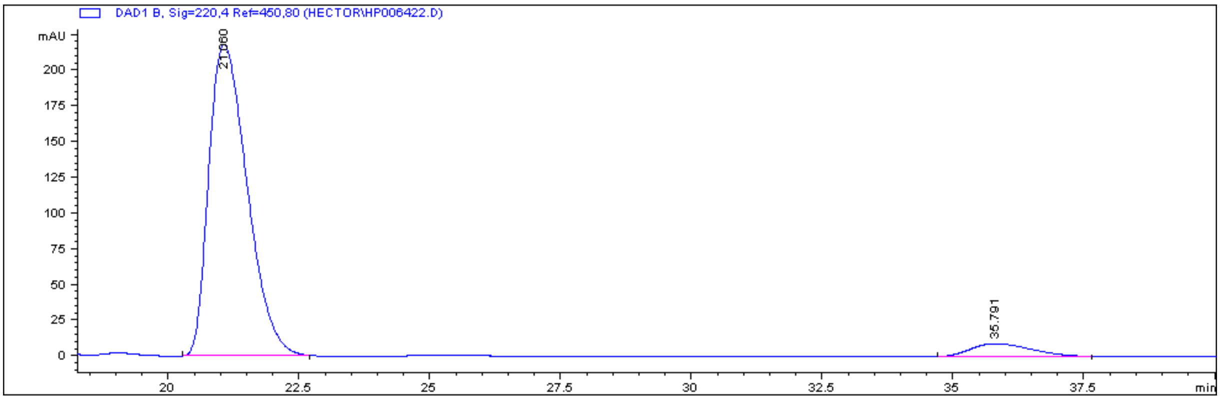

Peak Name	Time	Area	Height	Area%	Symmetry
1	21.06	11152.6	218.3	93.859	0.631
2	35.791	729.7	9.1	6.141	0.638

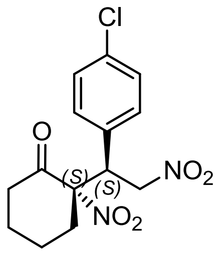

7ba

Racemic molecule:

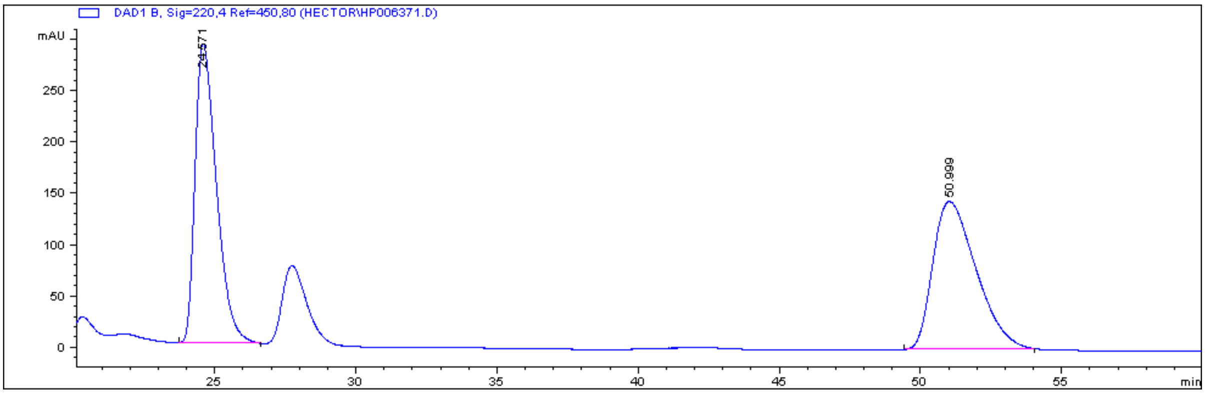

Peak Name	Time	Area	Height	Area%	Symmetry
1	24.571	15376.7	291.4	50.073	0.581
2	50.999	15332.1	144	49.927	0.593

HPLC profile for entry 9, table 4. 100:0 rd, 91:9 er.

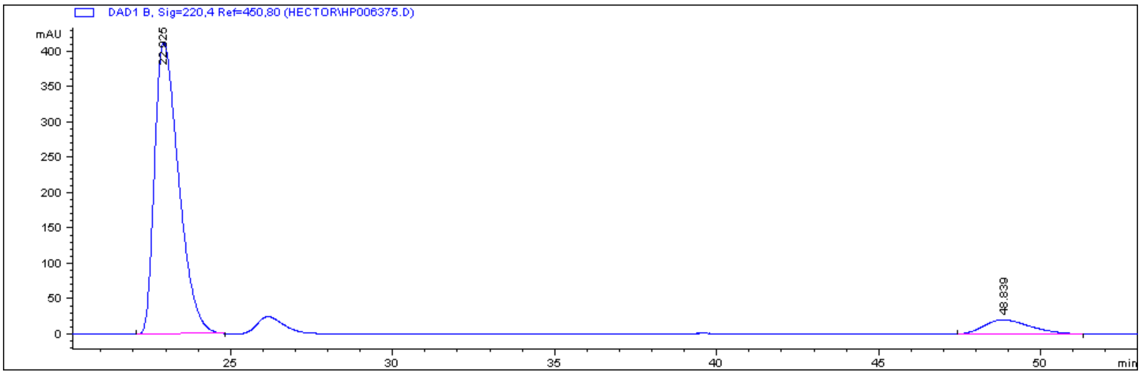

Peak Name	Time	Area	Height	Area%	Symmetry
1	22.925	21353	413.7	91.382	0.571
2	48.839	2013.6	20.2	8.618	0.704

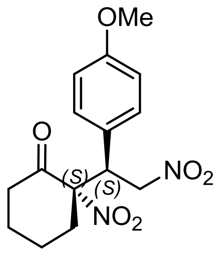

7da

Racemic molecule:

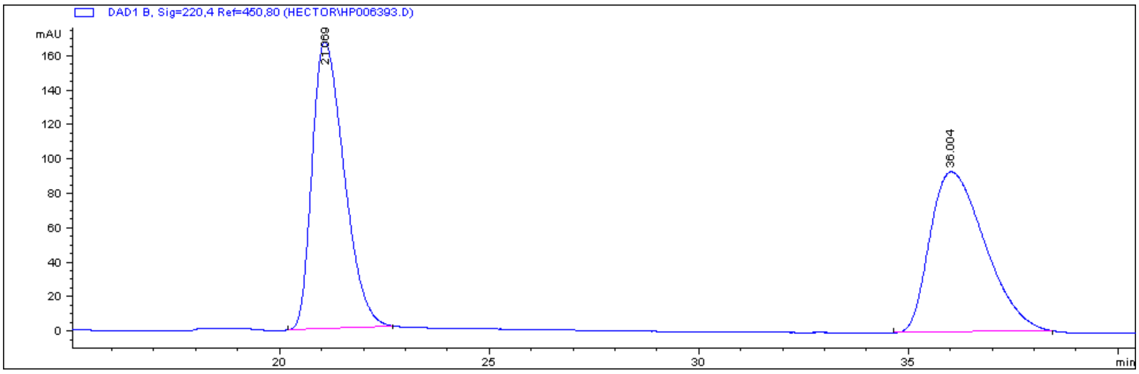

Peak Name	Time	Area	Height	Area%	Symmetry
1	21.069	8883.6	167.1	50.950	0.625
2	36.004	8552.3	93.4	49.050	0.593

HPLC profile for entry 11, table 4. 100:0 rd, 89:11 er.

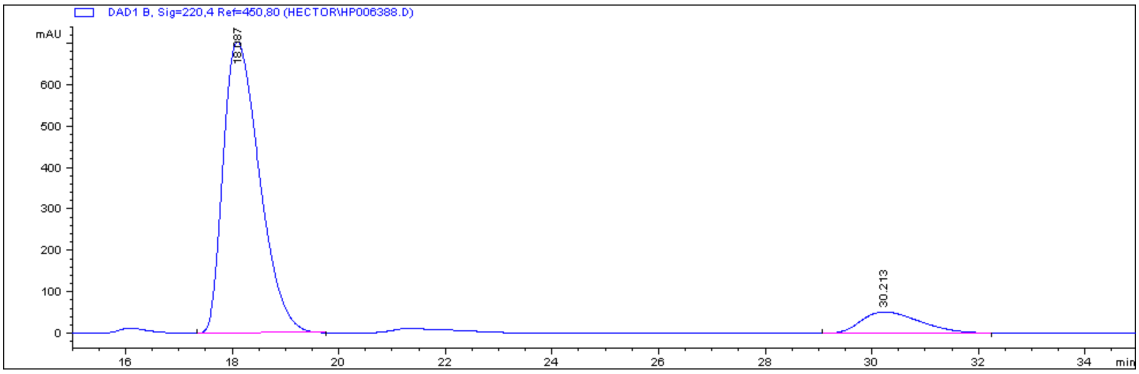

Peak Name	Time	Area	Height	Area%	Symmetry
1	18.087	33190	703.8	89.299	0.601
2	30.213	3977.2	51.7	10.701	0.609

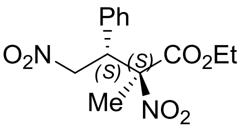

7ab

Racemic molecule:

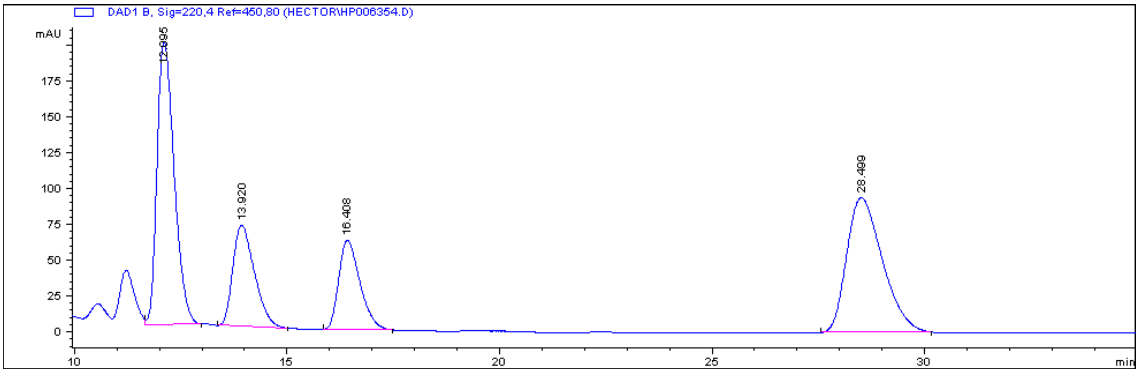

Peak Name	Time	Area	Height	Area%	Symmetry
1	12.095	5540.7	197.7	35.181	0.662
2	13.92	2507	70.6	15.918	0.649
3	16.408	2174.6	62.4	13.808	0.652
4	28.499	5527	94.2	35.094	0.676

HPLC profile for entry 15, table 4. 76:24 rd, 74:26 er.

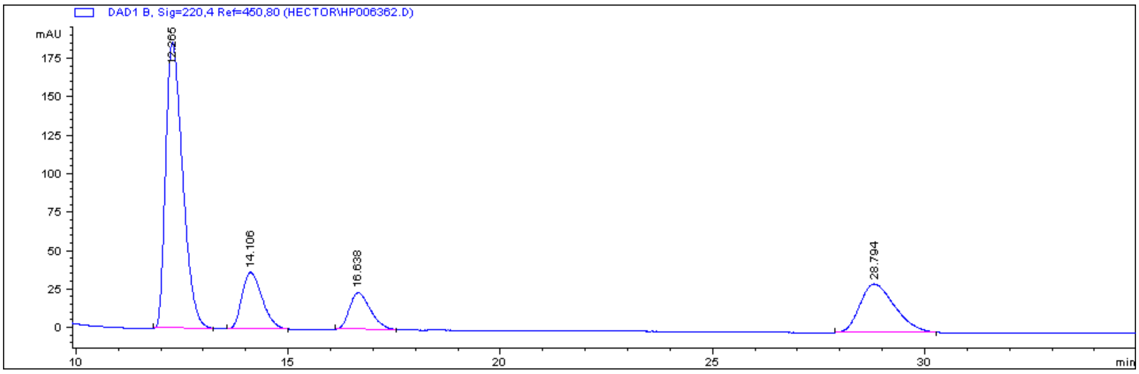

Peak Name	Time	Area	Height	Area%	Symmetry
1	12.265	5182.3	186.5	57.326	0.625
2	14.106	1220.8	37	13.504	0.705
3	16.638	829.1	24.1	9.172	0.659
4	28.794	1807.9	31.6	19.999	0.65

4. REFERENCES

1. Okino, T.; Hoashi, Y.; Furukawa, T.; Xu, X.; Takemoto, Y. *J. Am. Chem. Soc.* **2005**, *127*, 119-125.

2. Barnes, D. M.; Ji, J.; Fickes, M. G.; Fitzgerald, M. A.; King, S. A.; Morton, H. E.; Plagge, F. A.; Preskill, M.; Wagaw, S. H.; Wittenberger, S. J.; Zhang, J. *J. Am. Chem. Soc*. **2002**, *124*, 13097-13105.

3. Wang, J.; Li, H.; Duan, W.; Zu, L.; Wang, W. *Org. Lett.* **2005**, *7*, 4713-4716.

4. Manzano, R.; Andrés, J. M.; Muruzabal, M. D.; Pedrosa, R. *Adv. Synth. Catal*. **2010**, *352*, 3364-3372.

5. Li, H.; Wang, Y.; Tang, L.; Wu, F.; Liu, X.; Guo, C.; Foxman, B. M.; Deng, L. *Angew. Chem. Int. Ed.* **2005**, *44*, 105-108.

6. P. Chauhan, P.; Chimni, S.S. *Asian J. Org. Chem*. **2012**, *1*, 138-141.

7. Jörres, M.; Schiffers, I.; Atodiresei, I.; Bolm. C. *Org. Lett*. **2012**, *14*, 4518-4521.

8. Li, H.; Wang, Y.; Tang, L.; Wu, F.; Liu, X.; Guo, C.; Foxman, B.M.; Deng, L. *Angew. Chem. Int. Ed.* **2005**, *44*, 105-108.
